# Supplementary material for: Chemical Characterization of 29 Industrial Hempseed (Cannabis sativa L.) Varieties
Source: Foods. 2024 Jan 9;13(2):210. doi: 10.3390/foods13020210 (PMC10814438; doi:10.3390/foods13020210)
Supplement: Supplementary file 1 [file foods-13-00210-s001.zip › foods-2748685-supplementary.pdf]

Figure S1. Gas Chromatography-Flame Ionization Detector chromatograms of fatty acids methyl esters of total fatty acids from 29 hempseed varieties

Antal

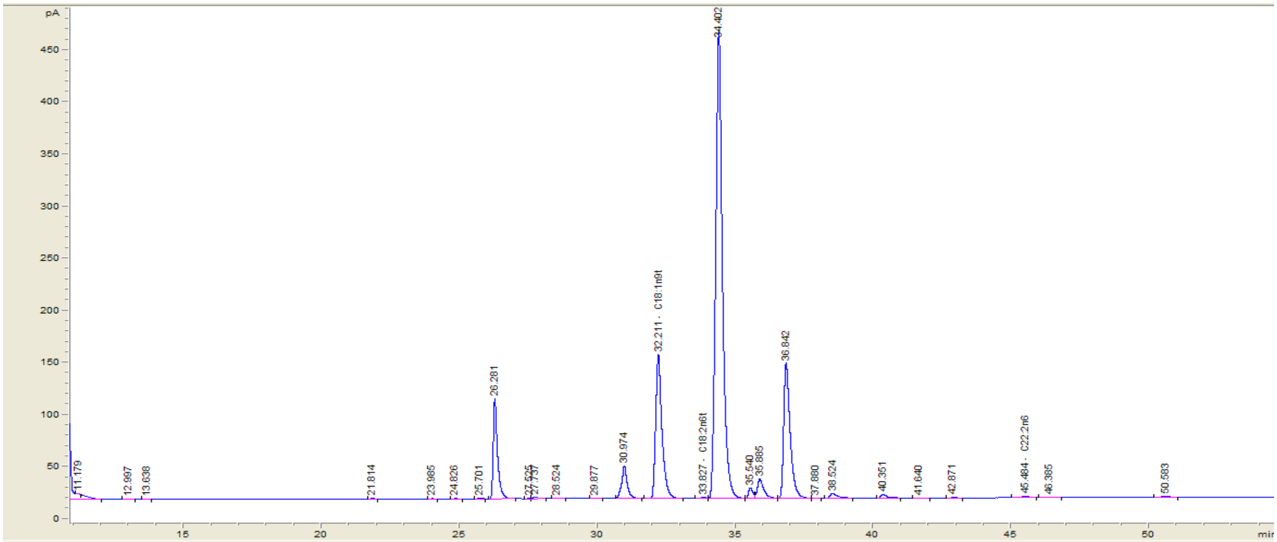

Bacalmas

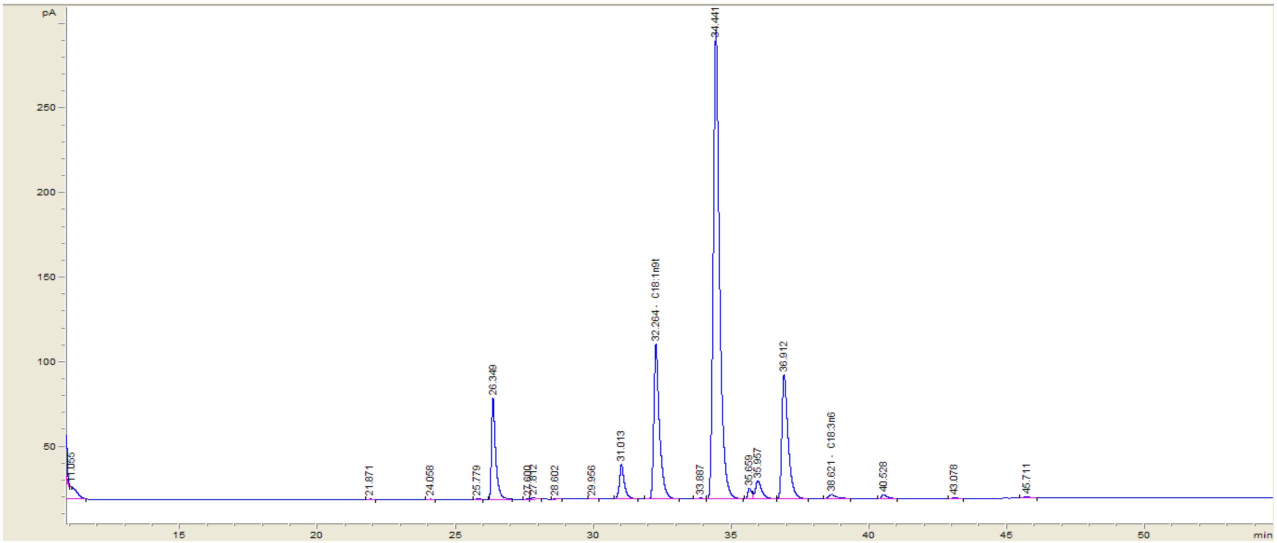

Carmagnola

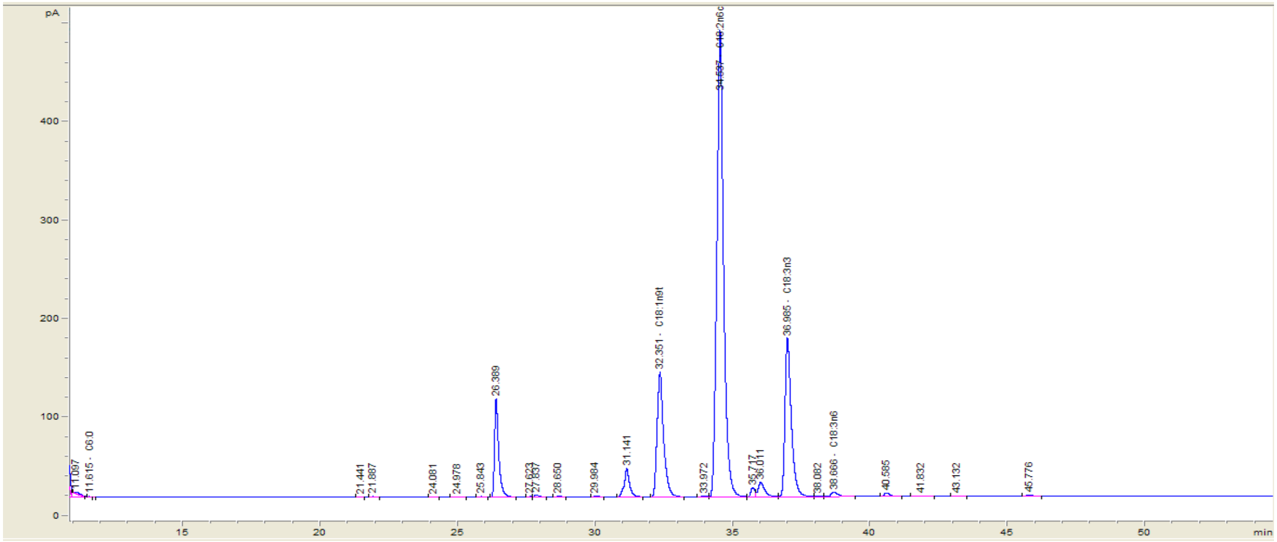

Chameleon

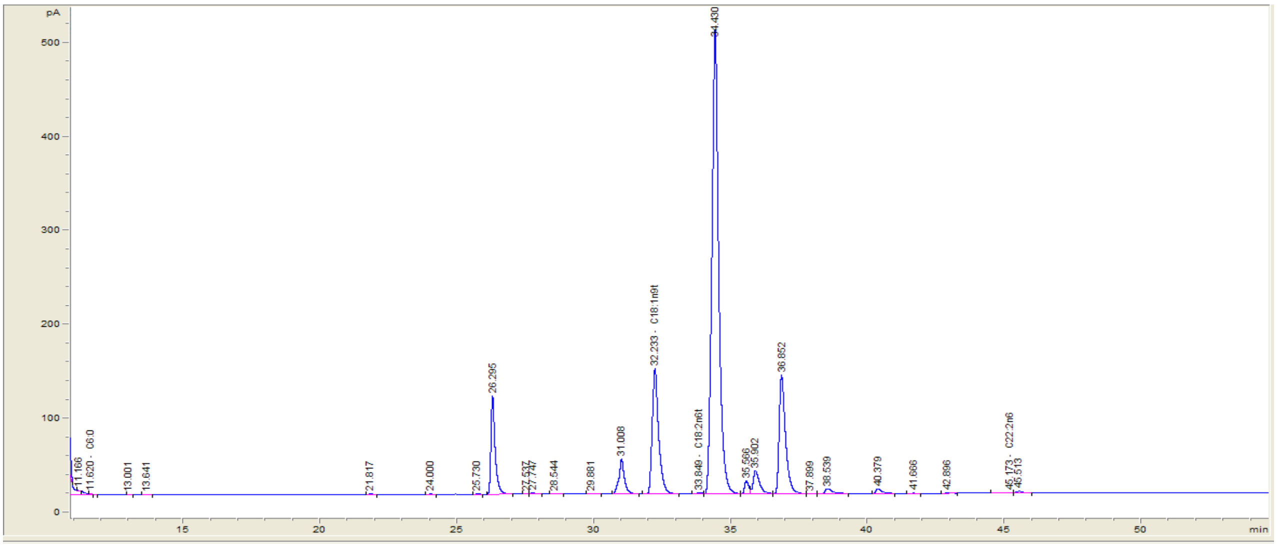

Dioica 88

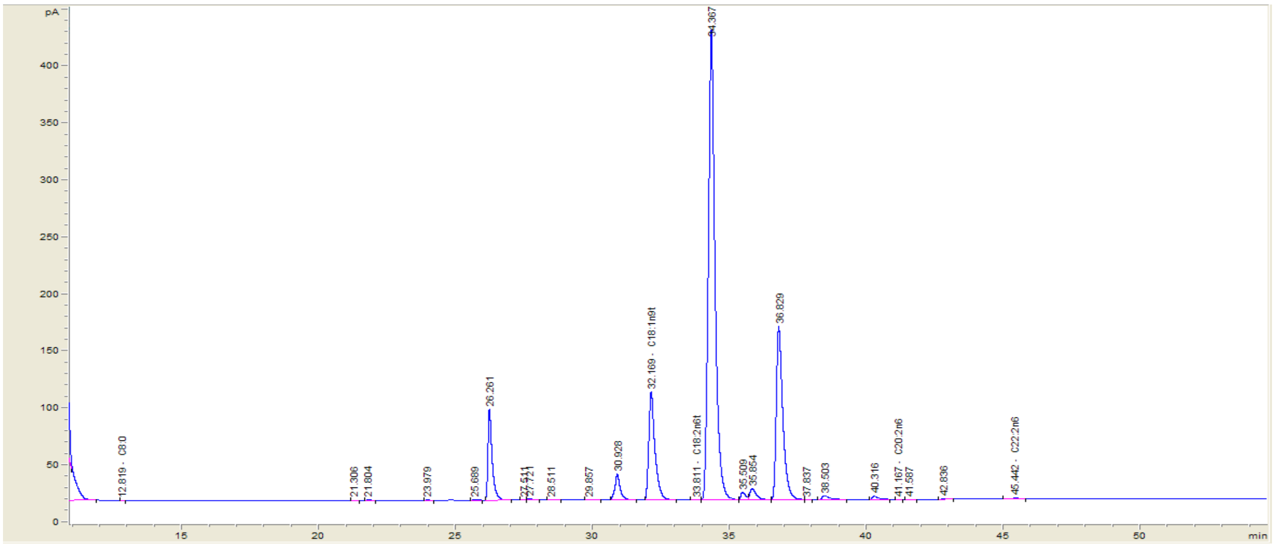

Epsilon 88

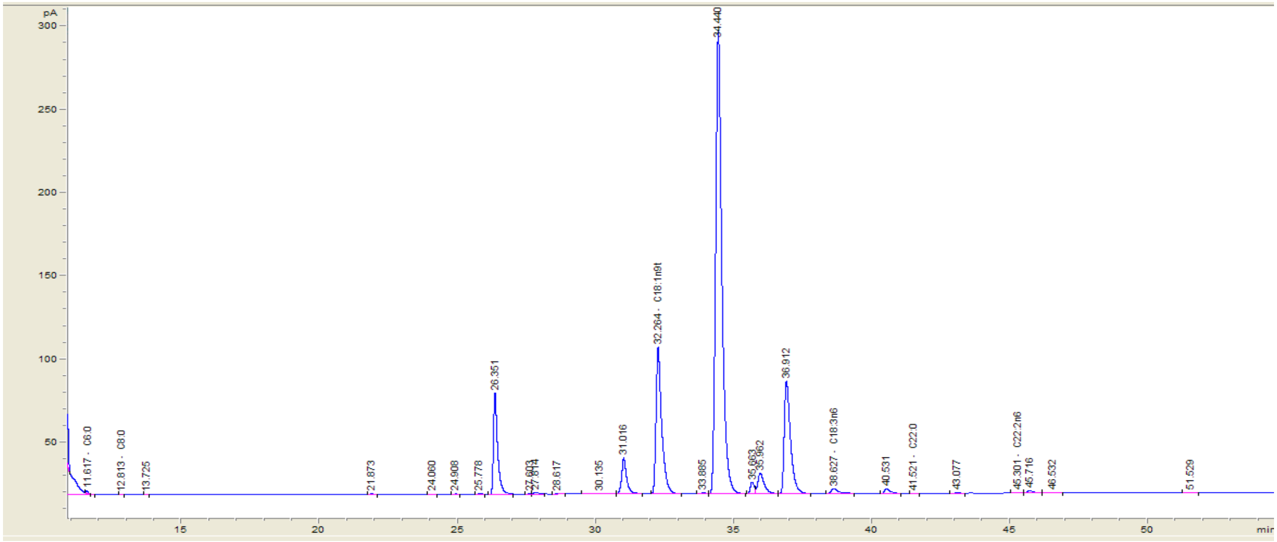

Fedora 17

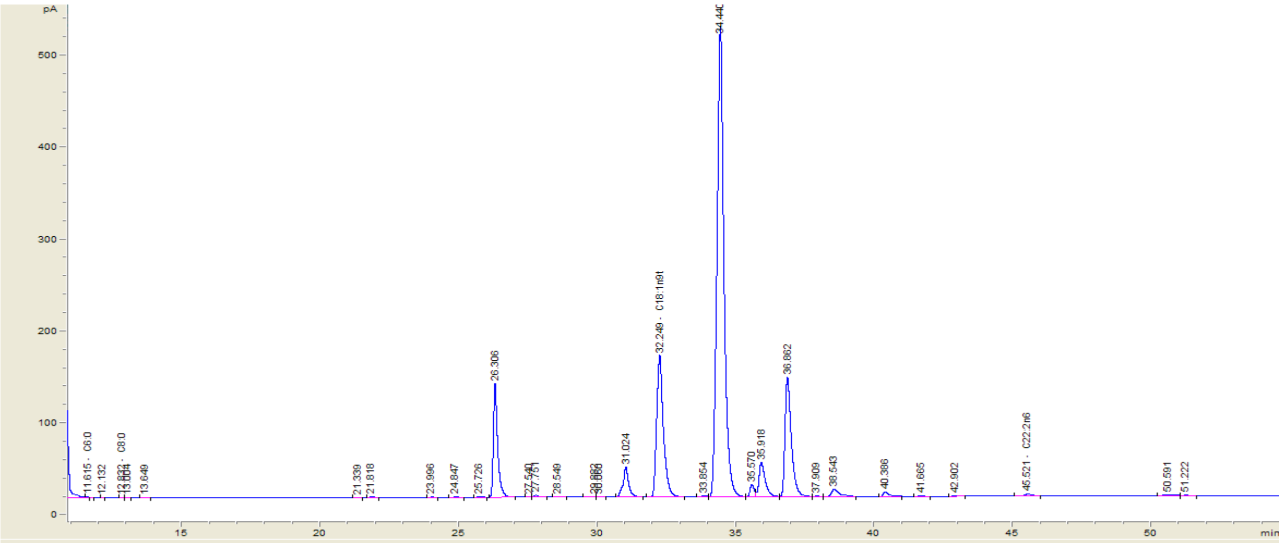

Felina 32

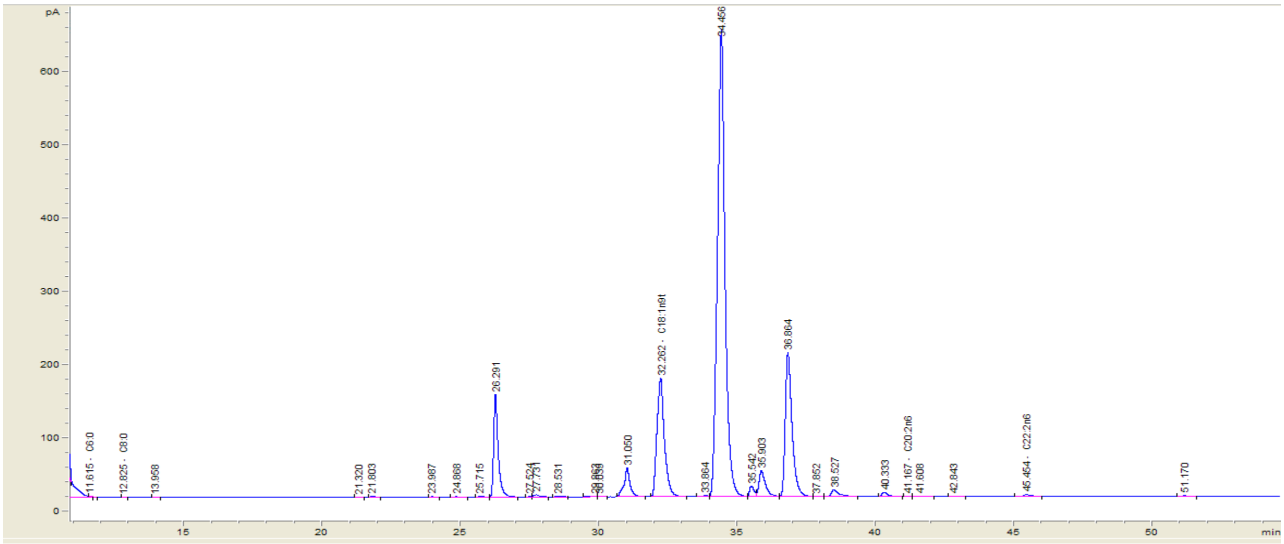

Ferimon FR 8194

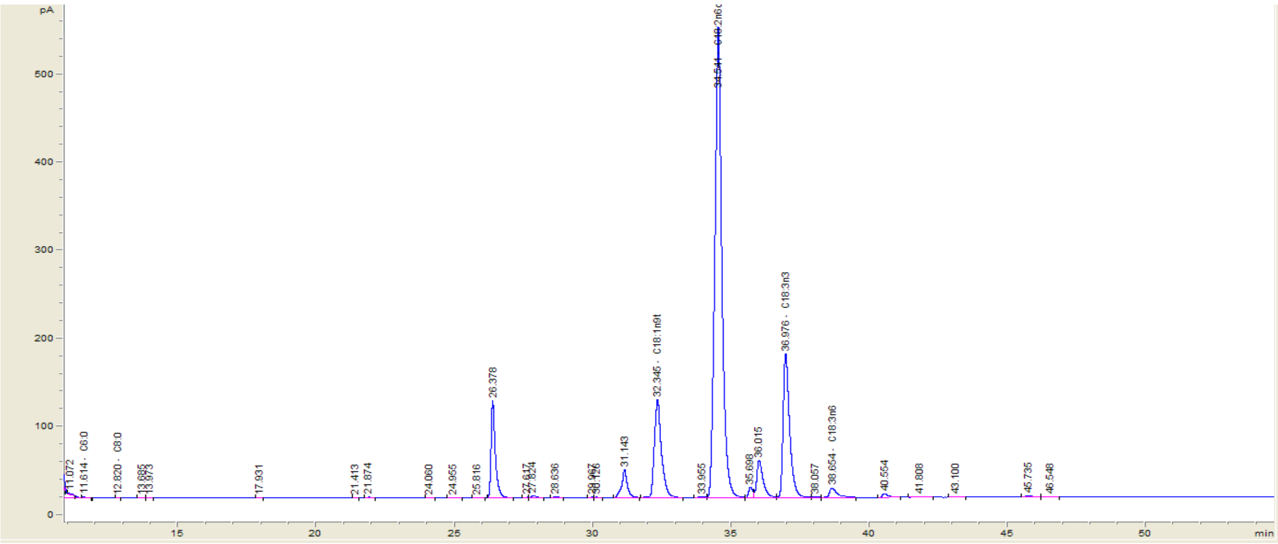

Fibrol

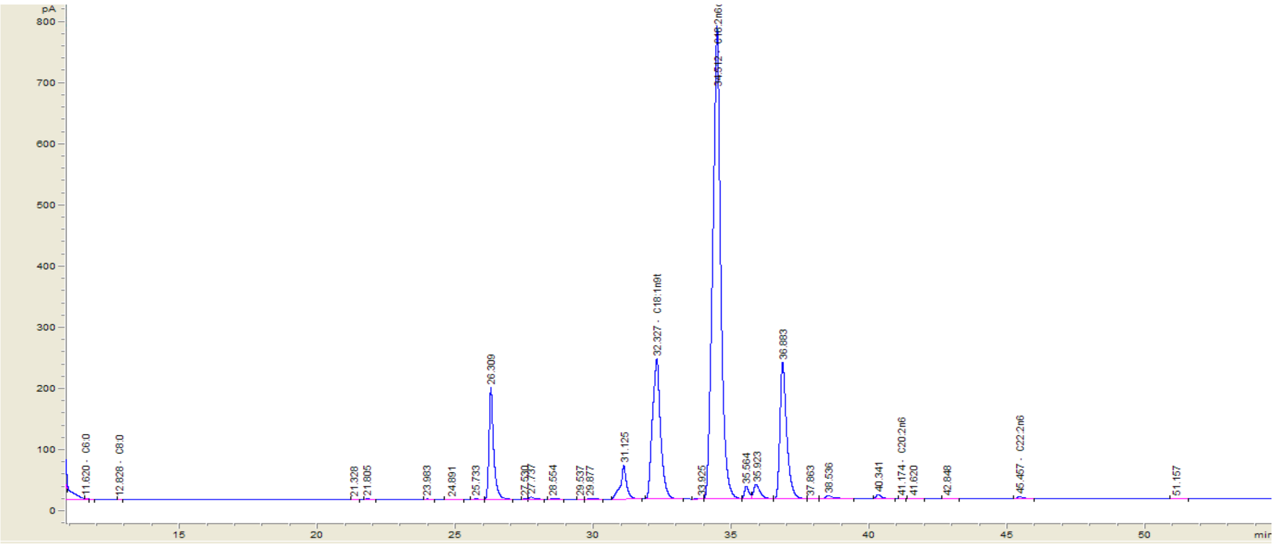

Futura 75

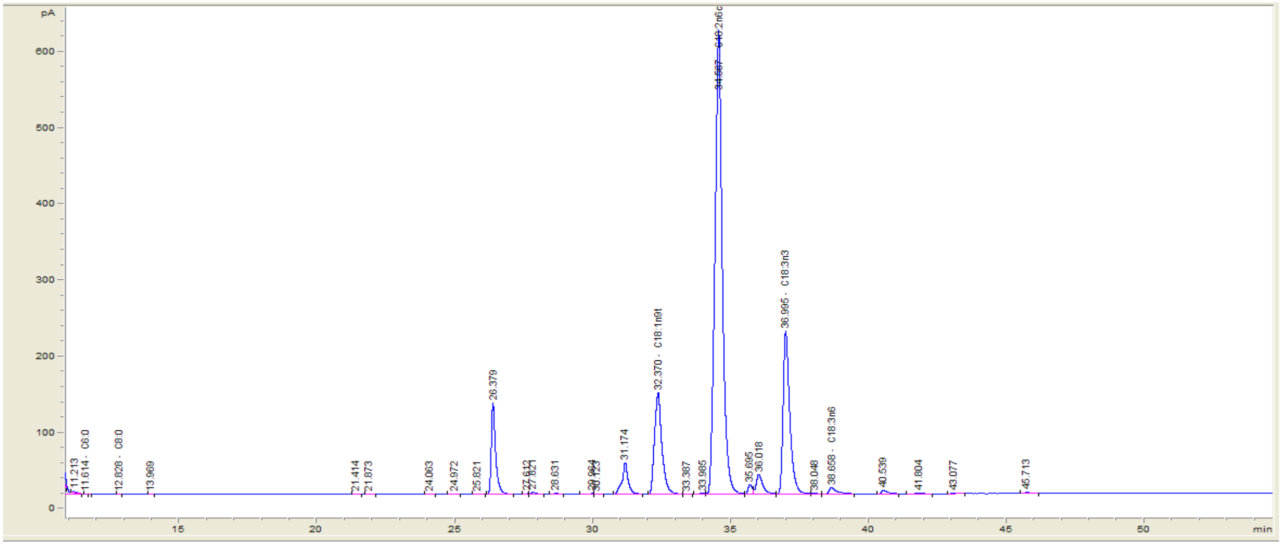

Helena

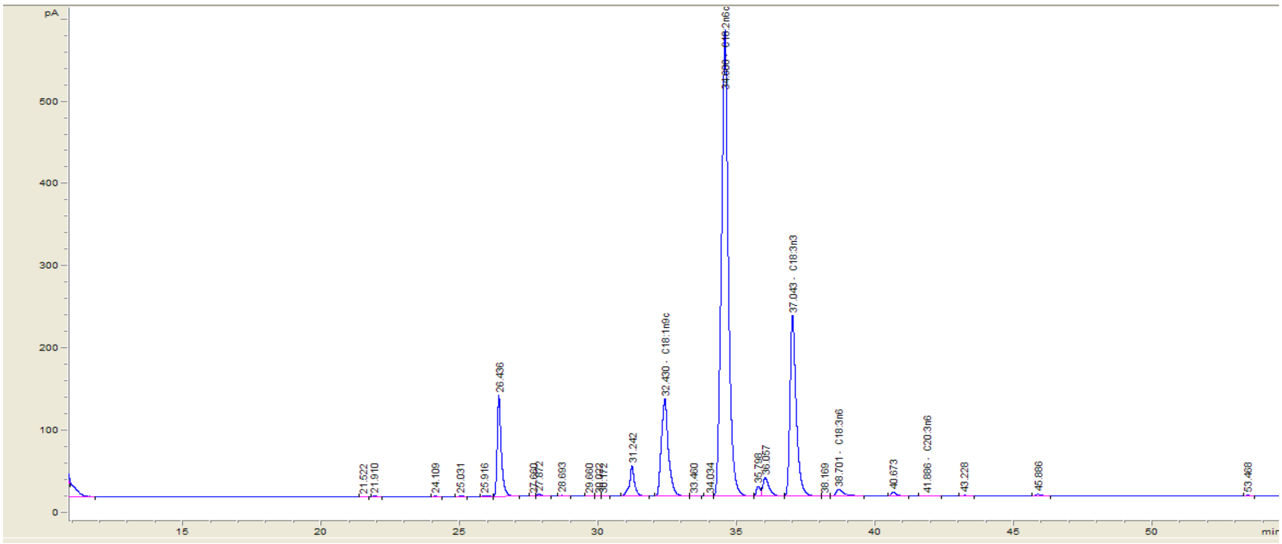

KC Dora

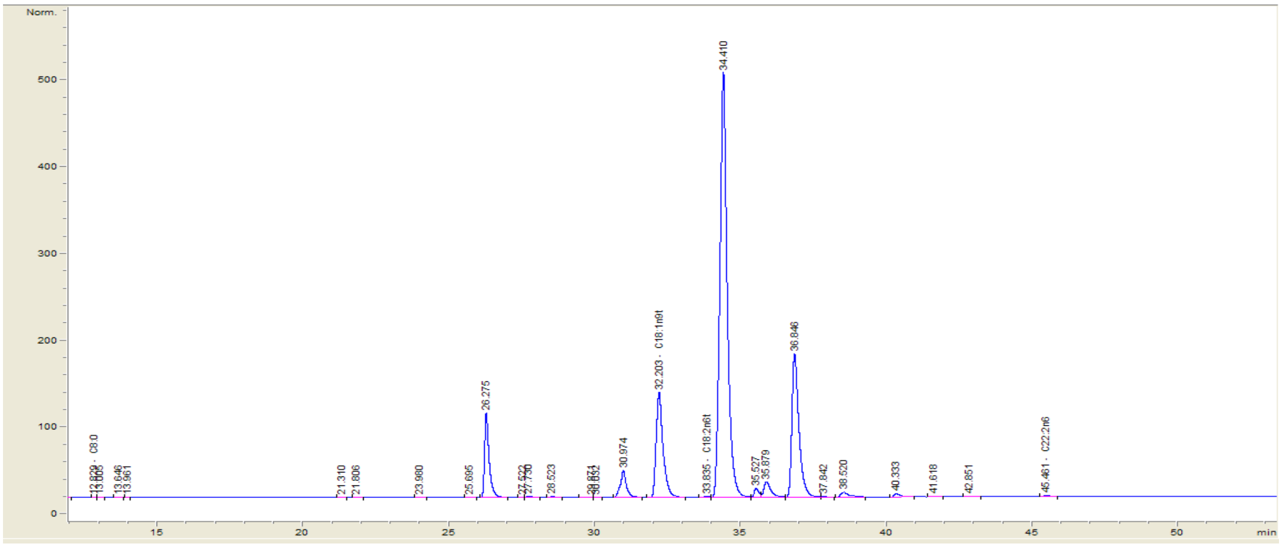

KC Virtus

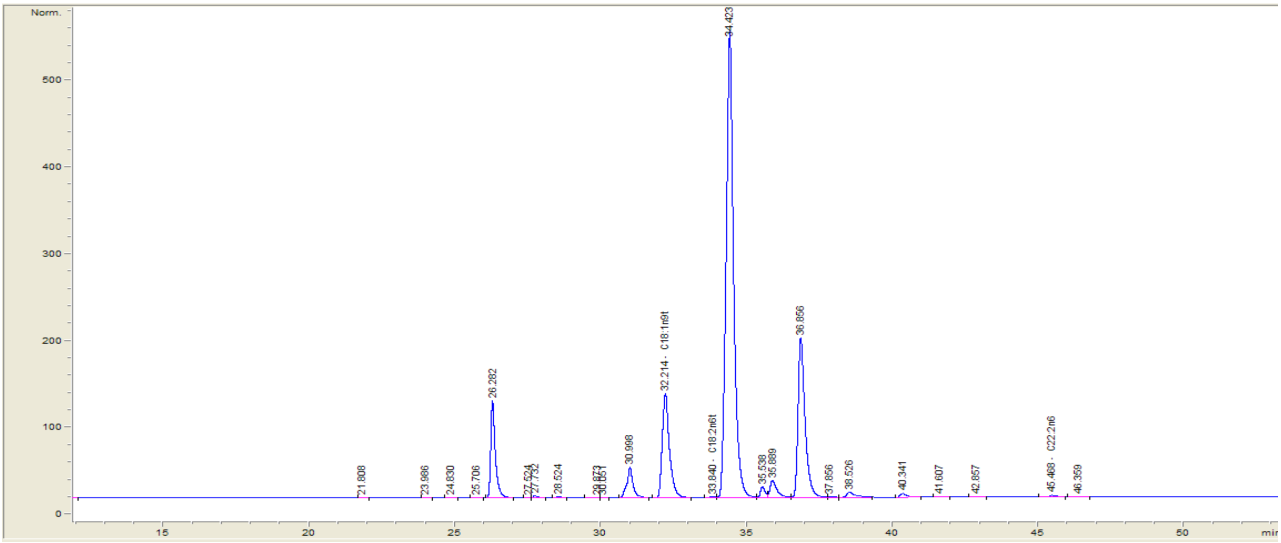

KC Zuzana

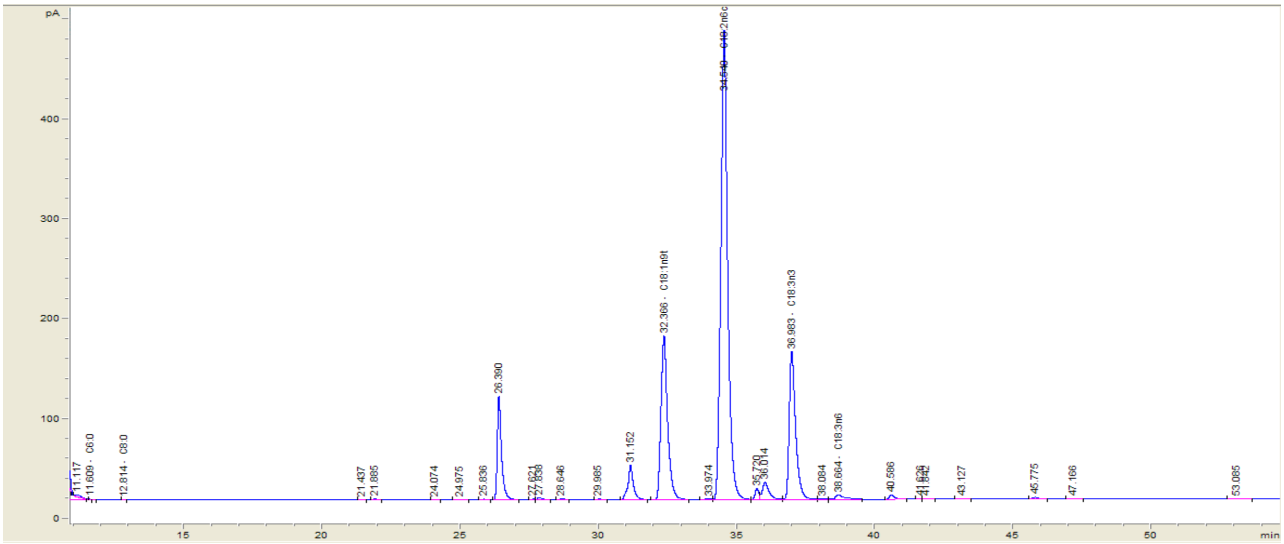

Kina

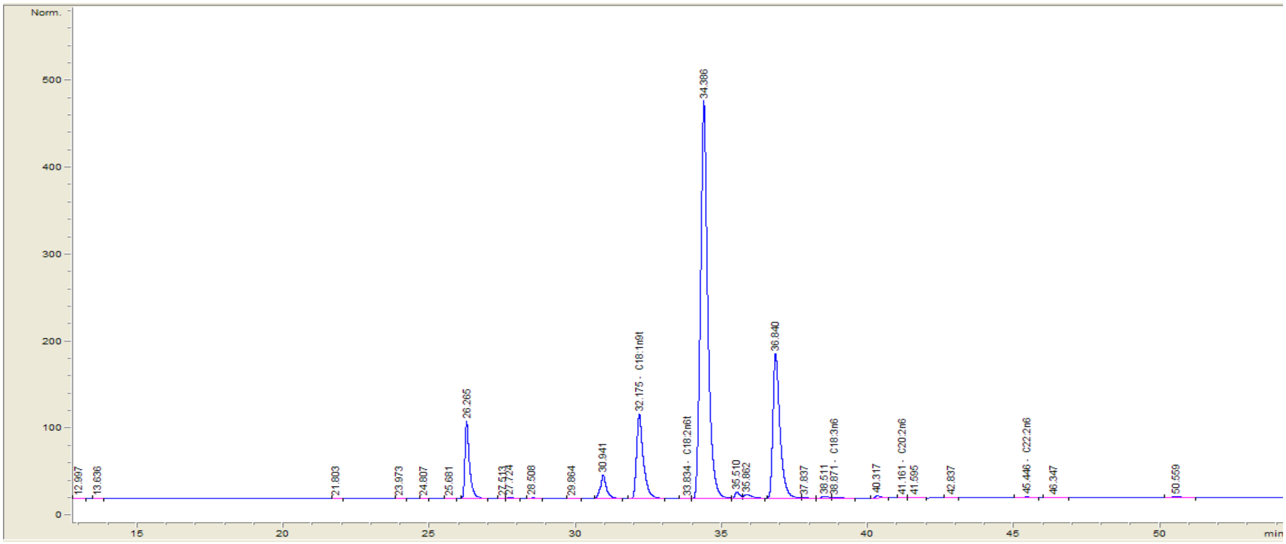

Kompolti

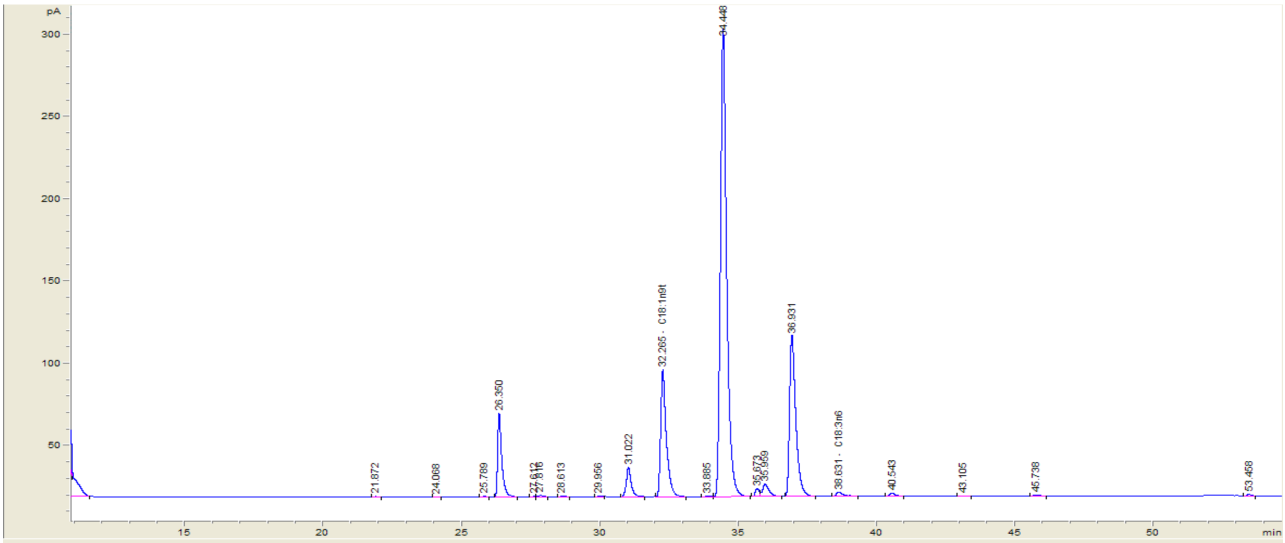

Lovrin110

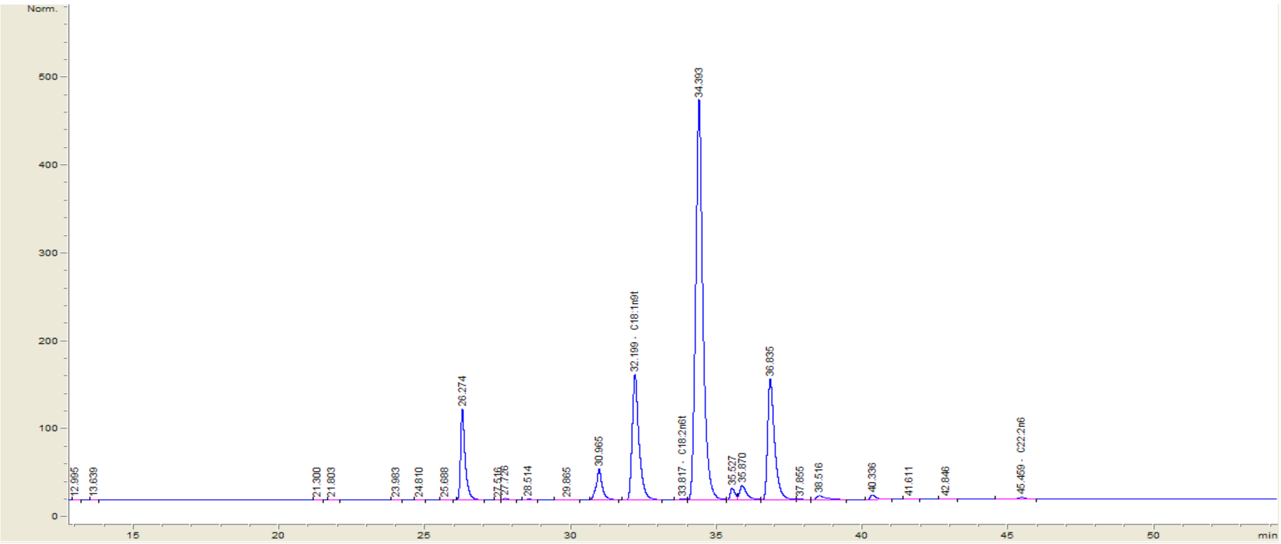

Marina

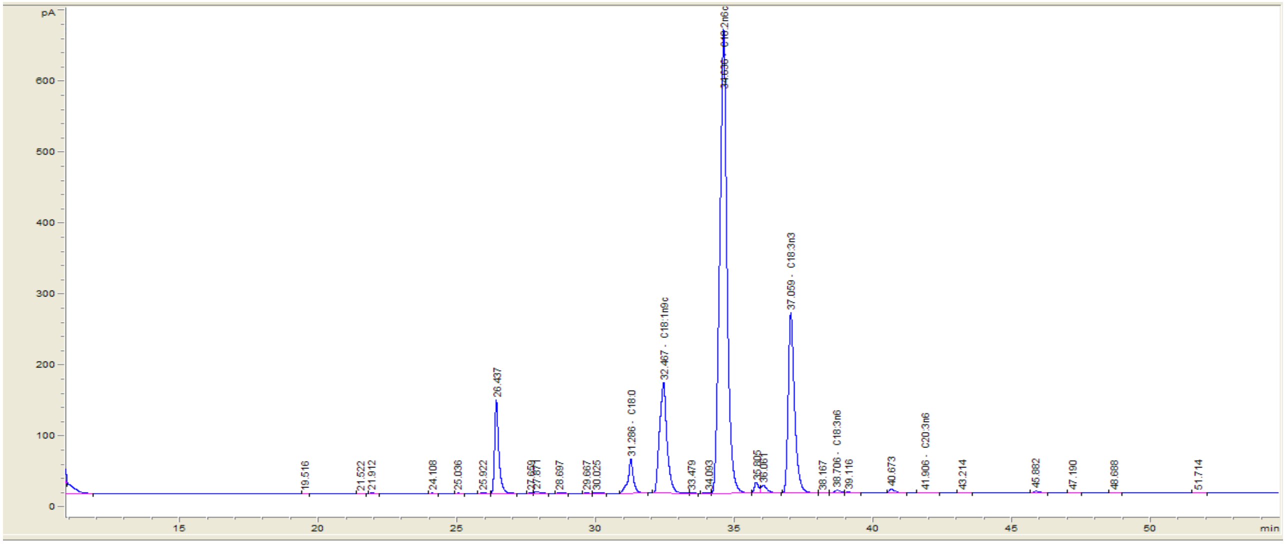

Monoica

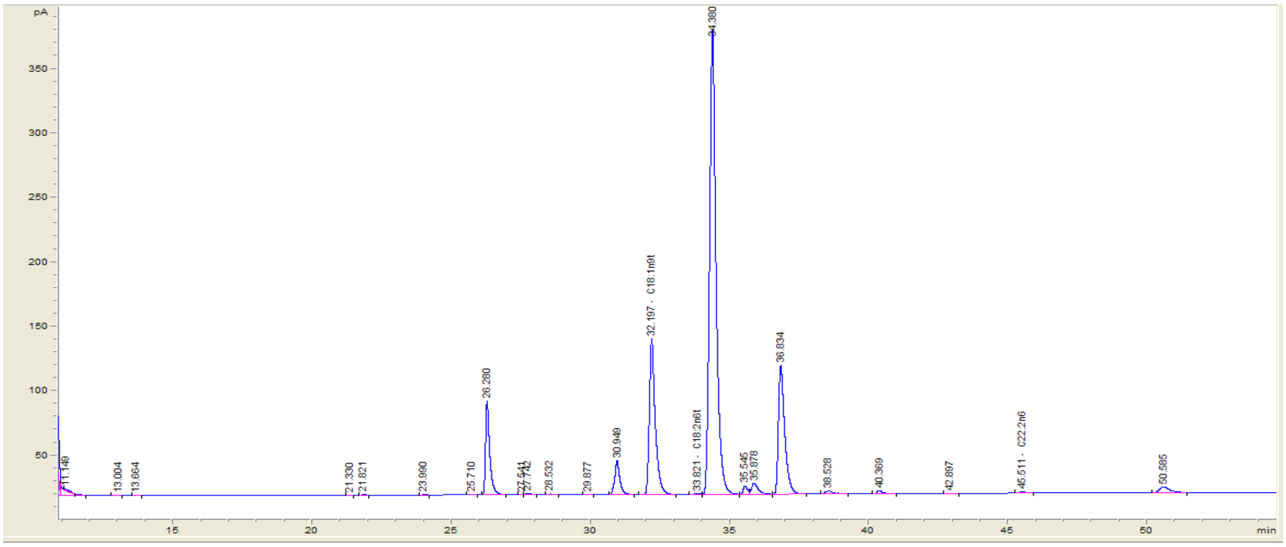

Novosadska

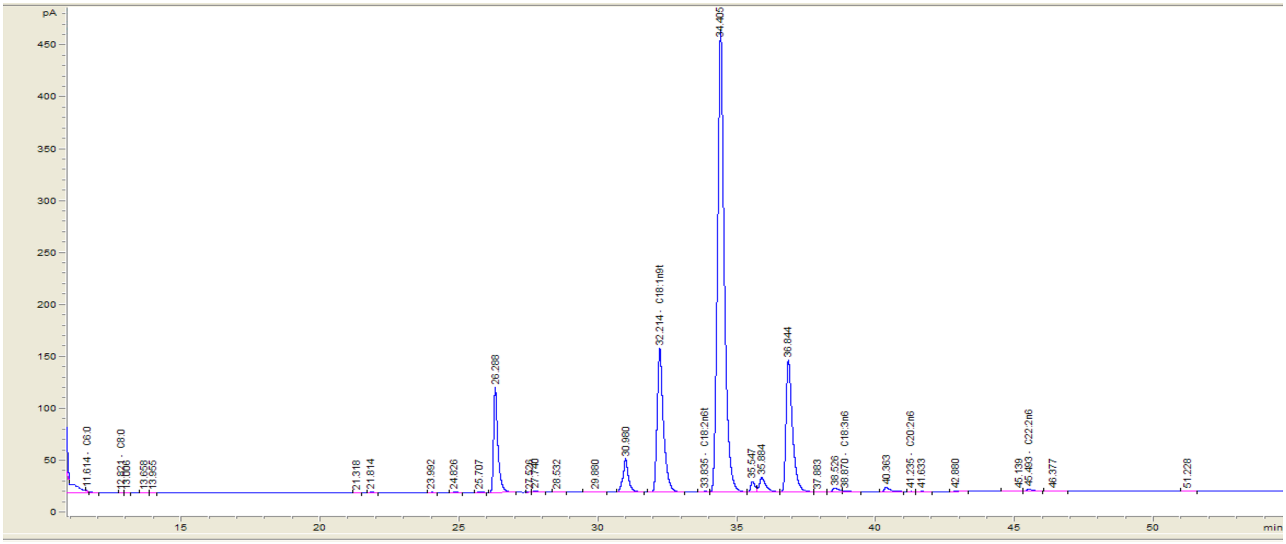

Novosadska+

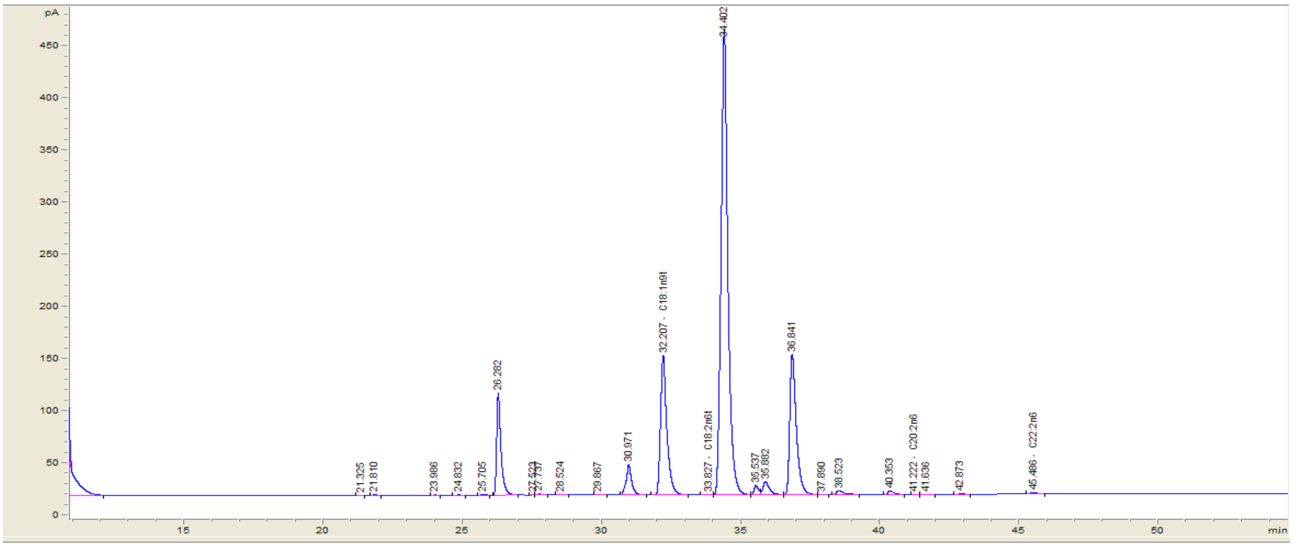

Santhica 23

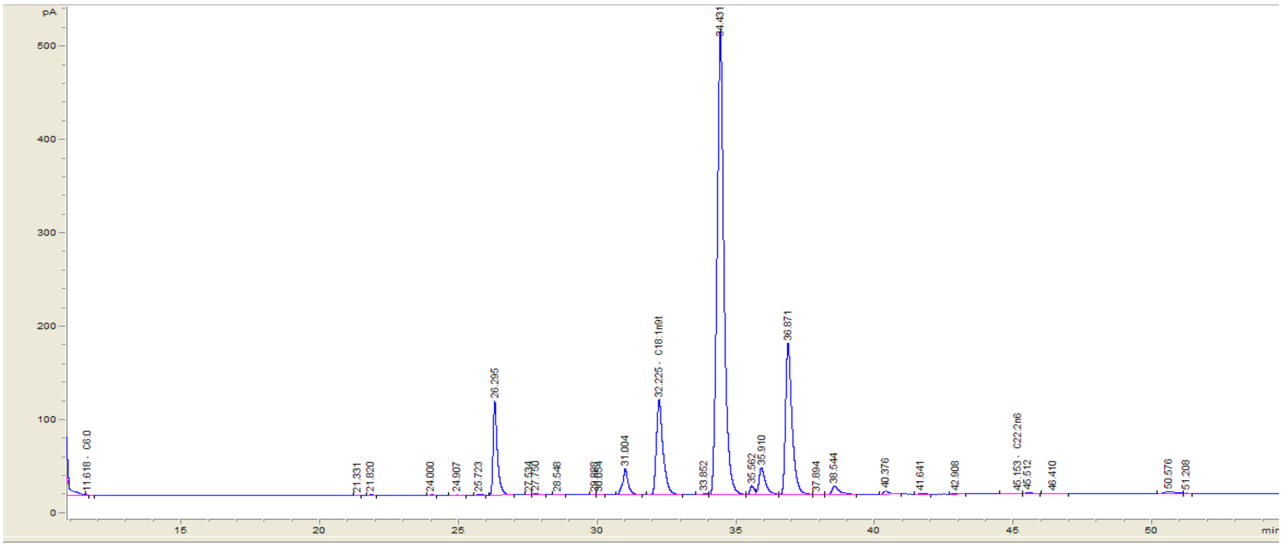

Secuieni jubileu

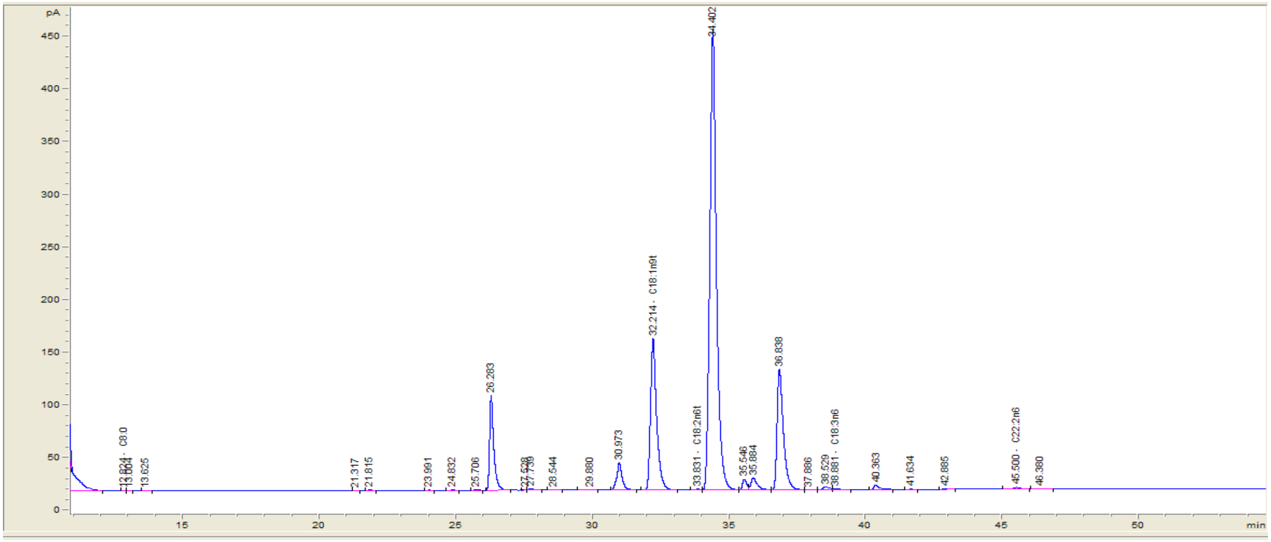

Silesia

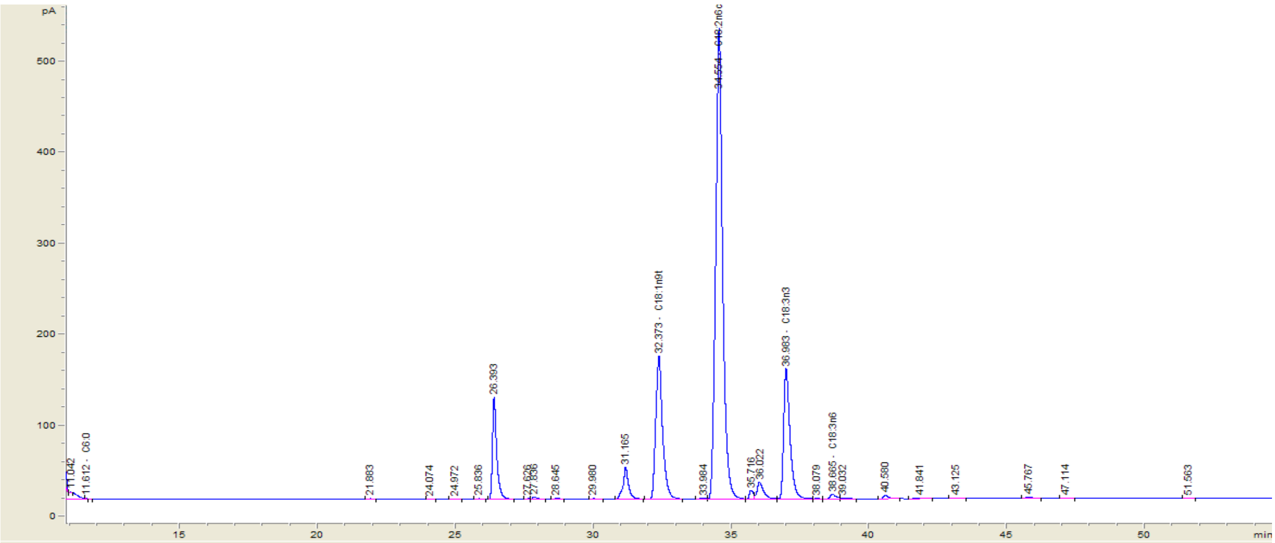

Simba

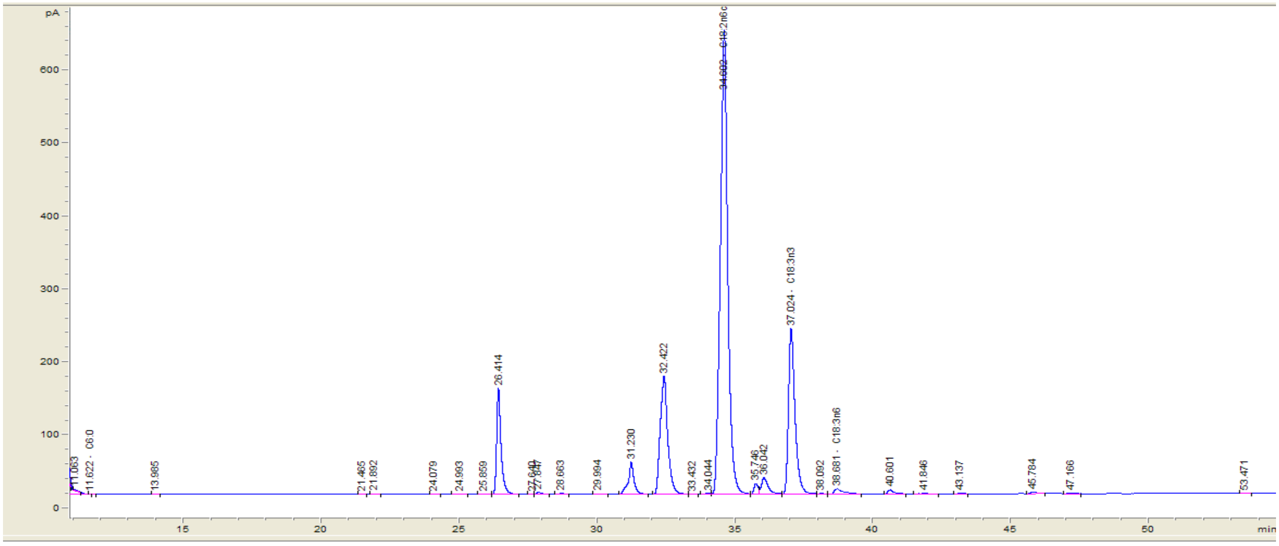

Tiborszallasi

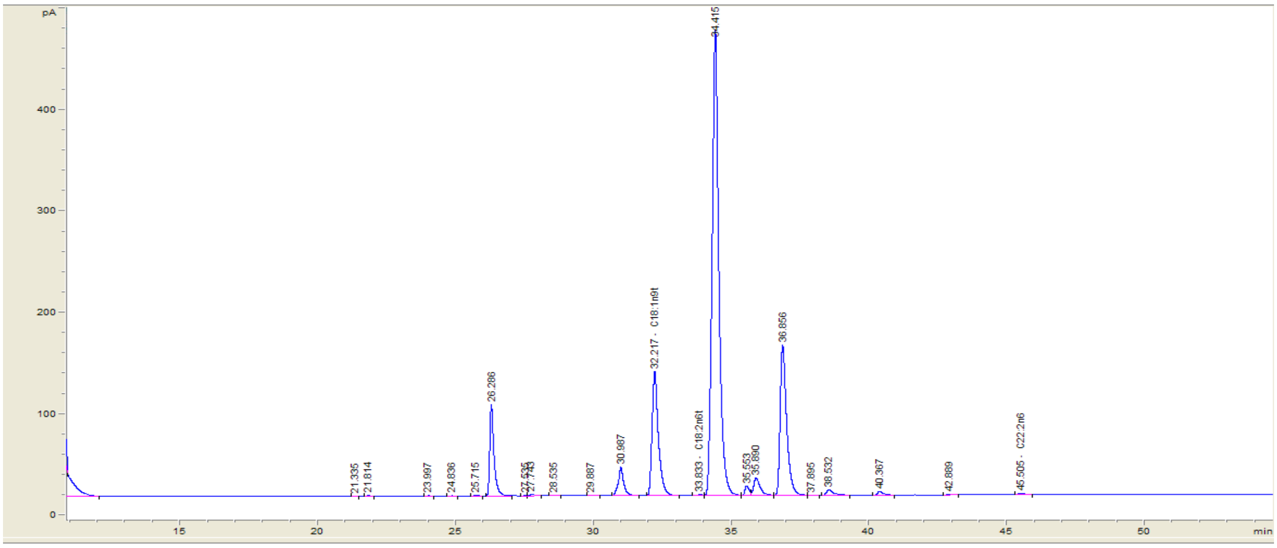

Tisza

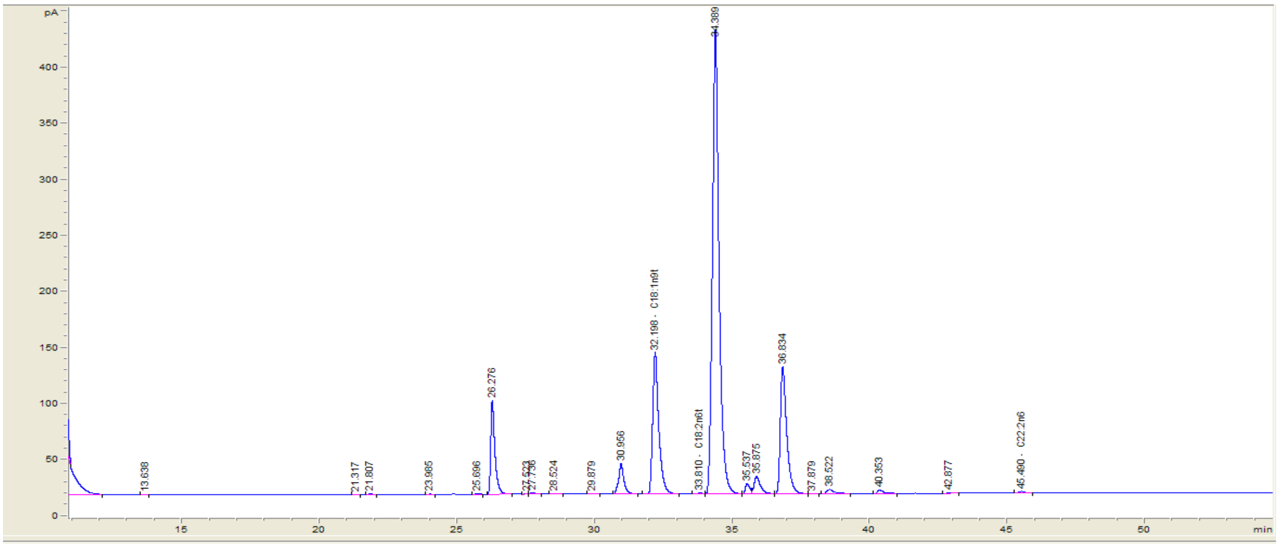

Wojko

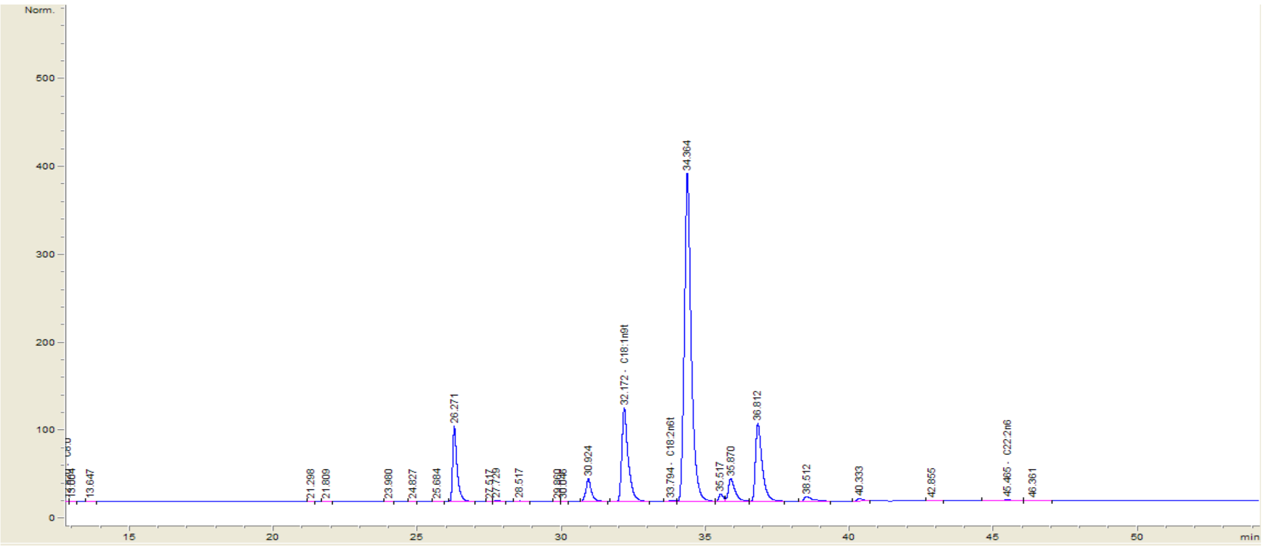

Figure S2. Ion exchange chromatograms of amino acids from 29 hempseed varieties

Antal

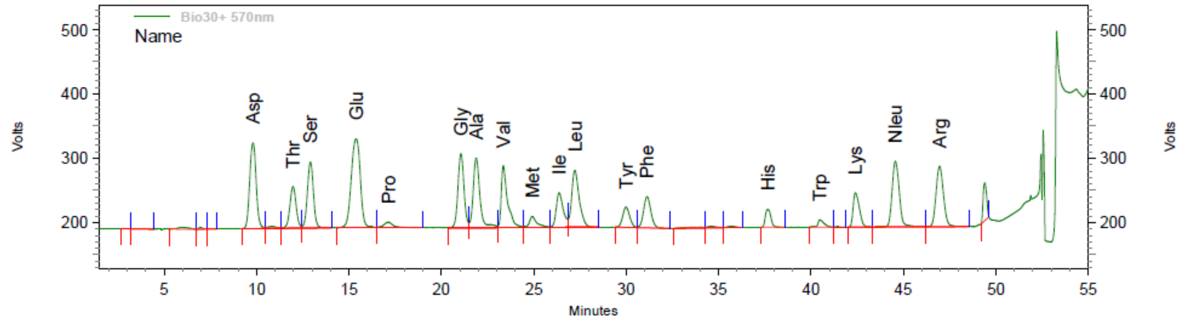

Bacalmas

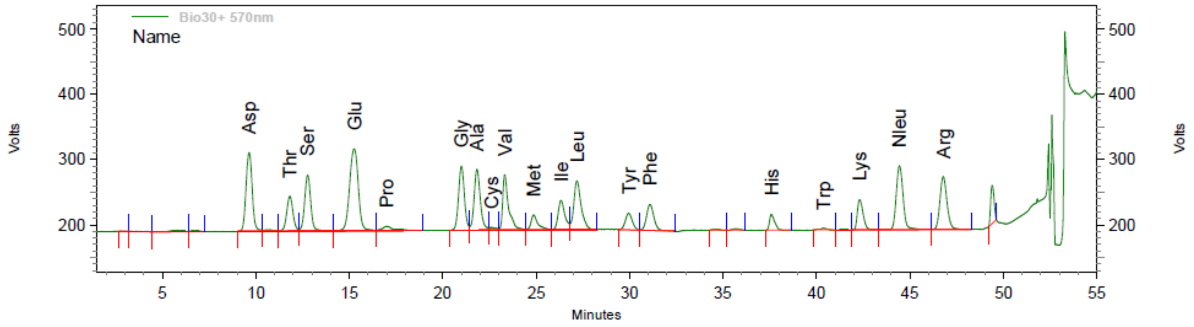

Carmagnola

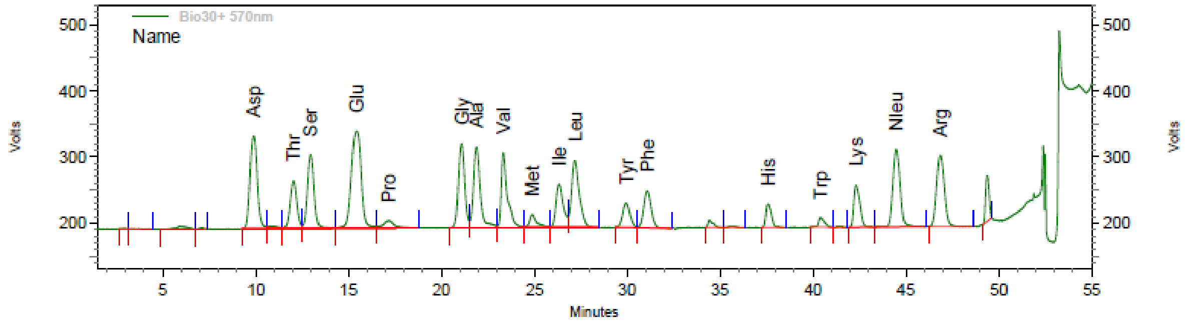

Chameleon

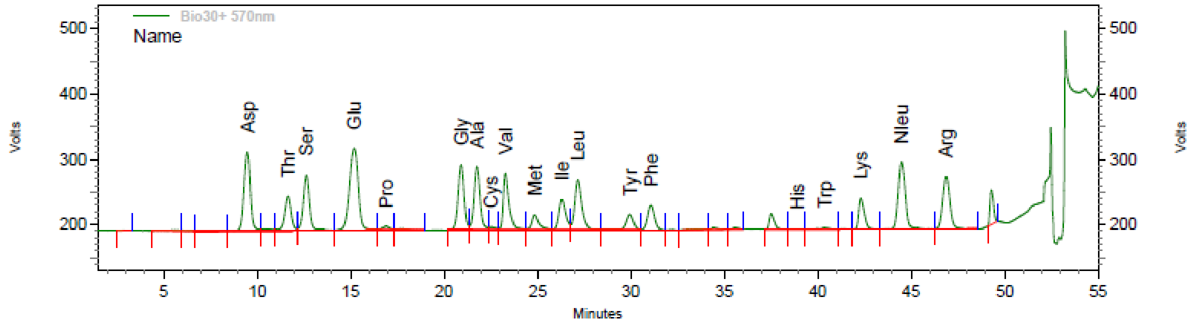

Dioica 88

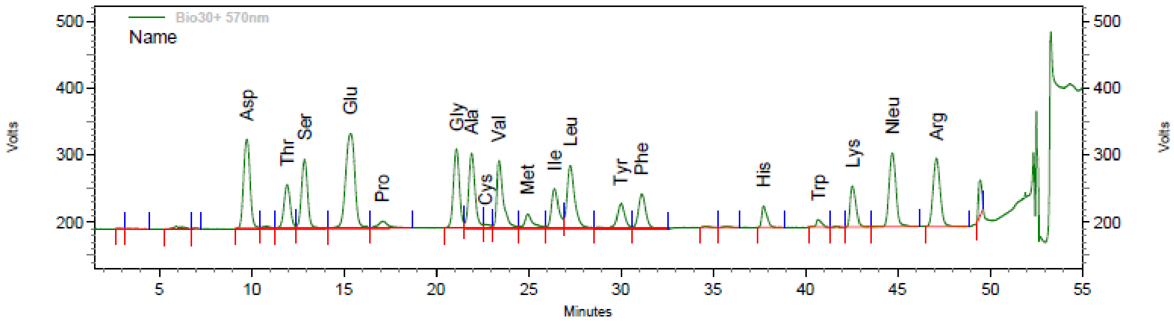

Epsilon 88

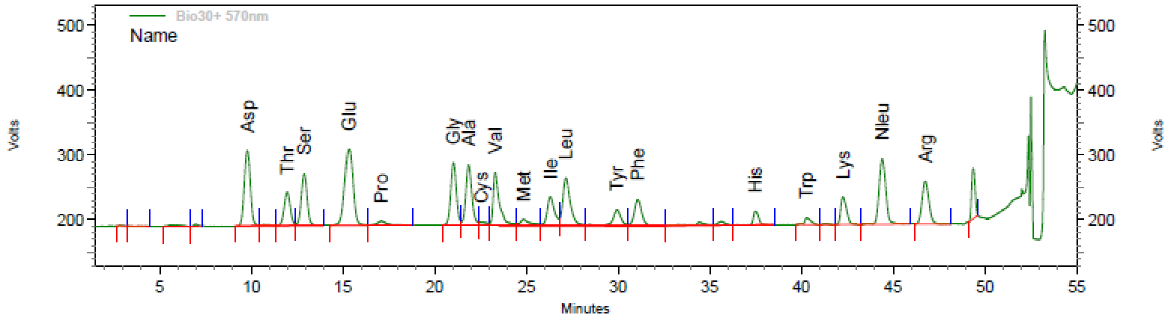

Fedora 17

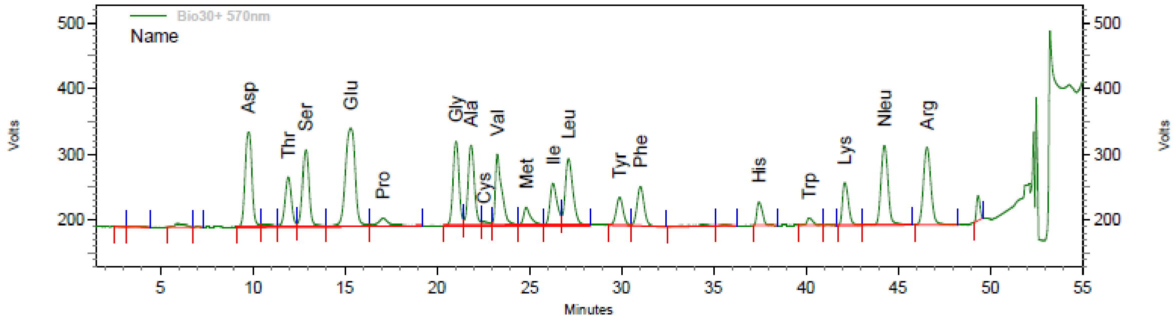

Felina 32

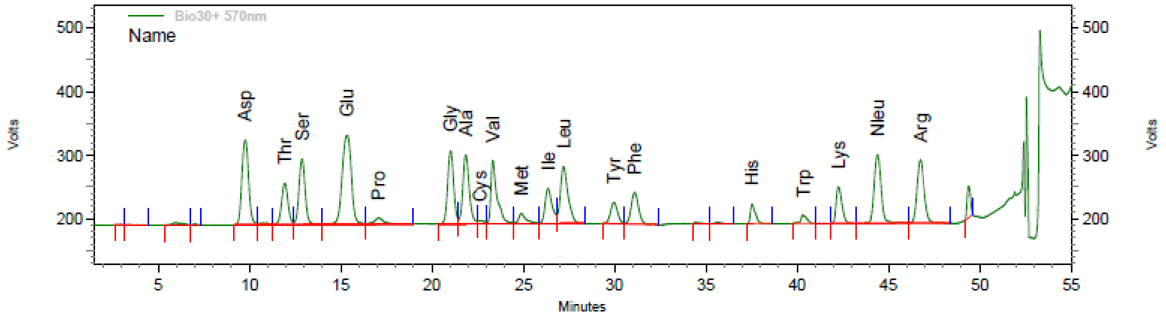

Ferimon FR 8194

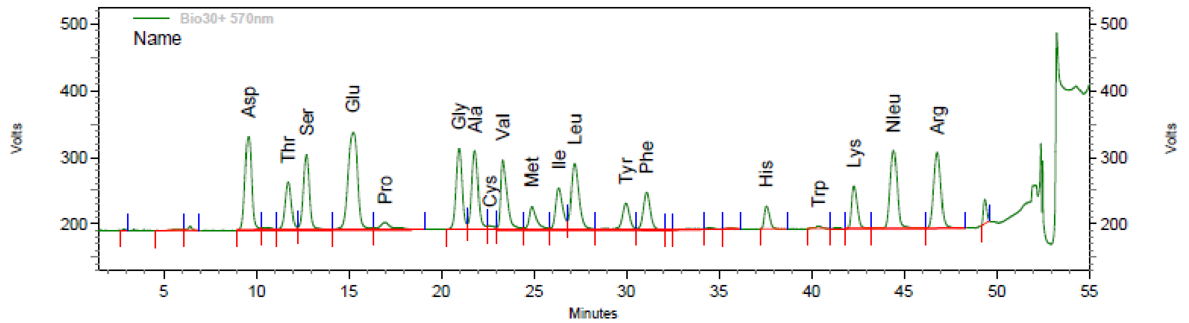

Fibrol

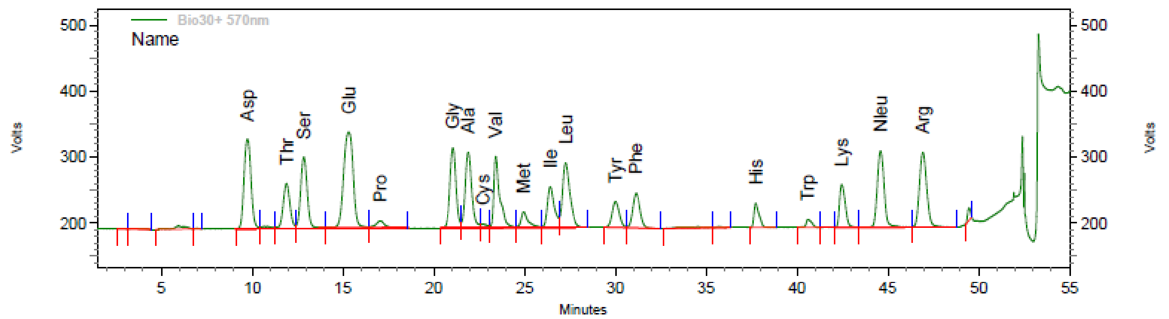

Futura 75

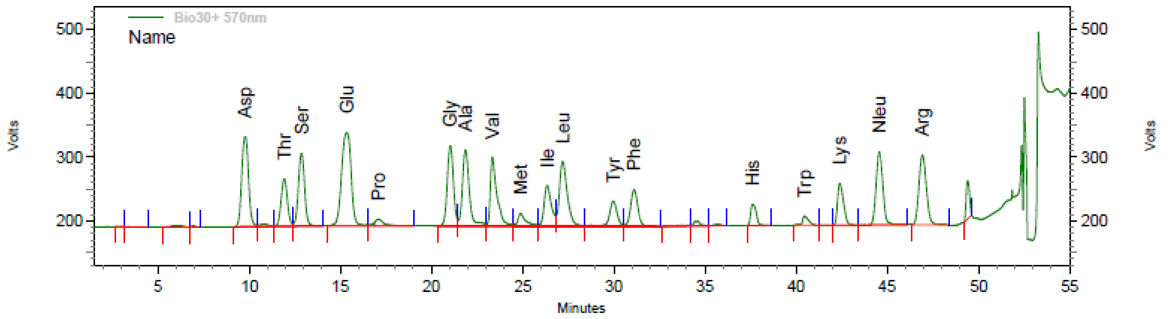

Helena

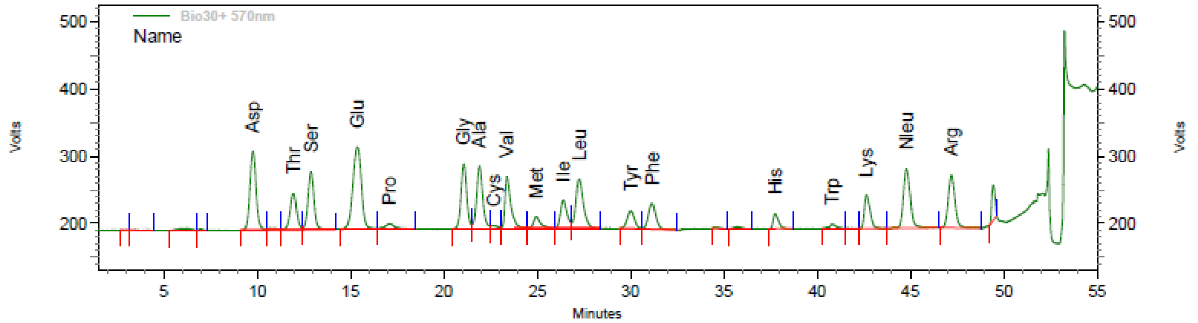

KC Dora

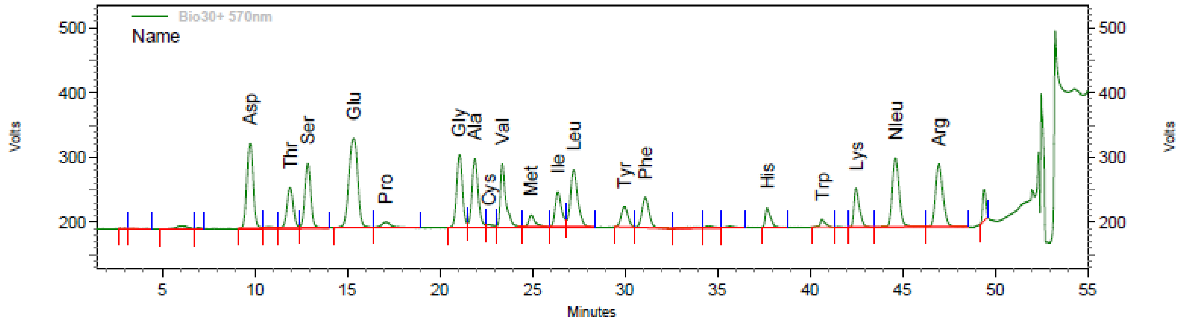

KC Virtus

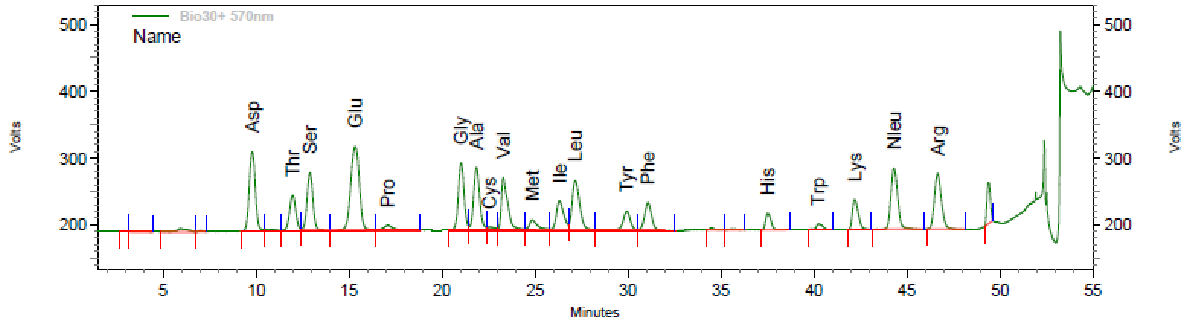

KC Zuzana

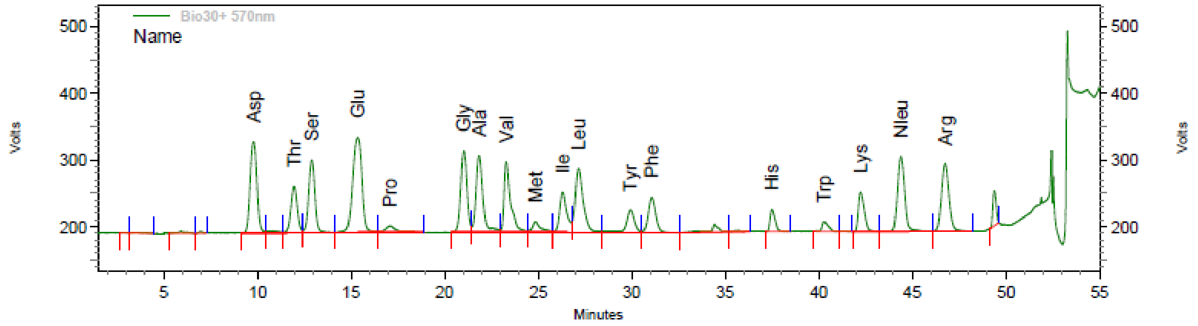

Kina

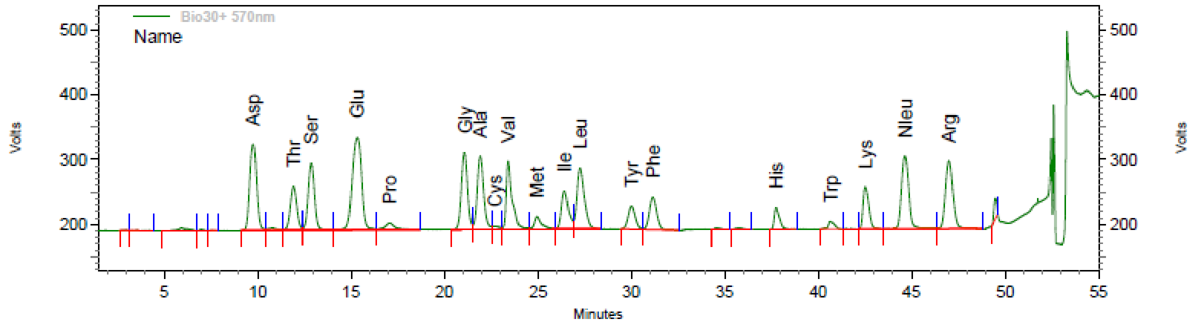

Kompolti

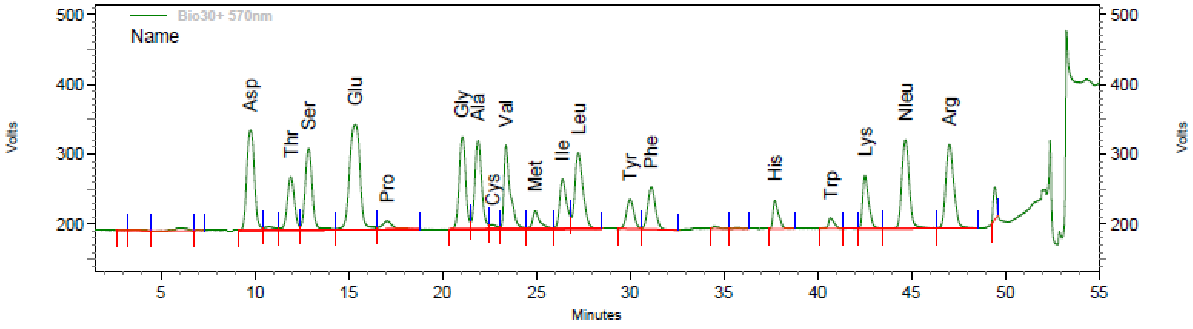

Lovrin110

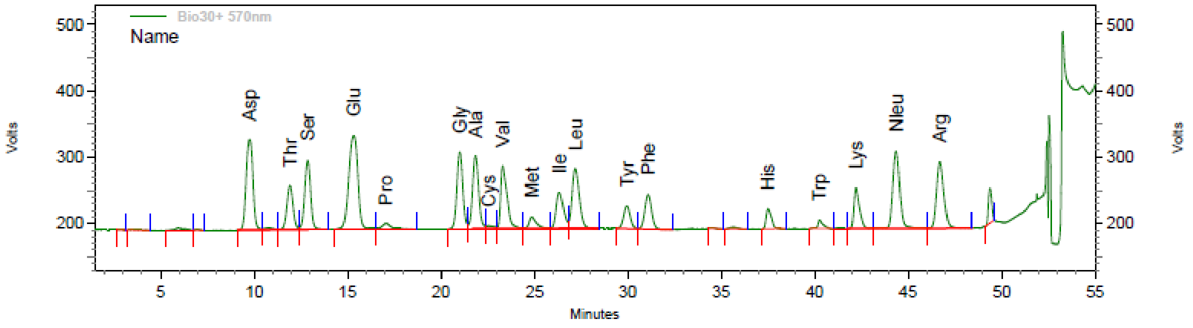

Marina

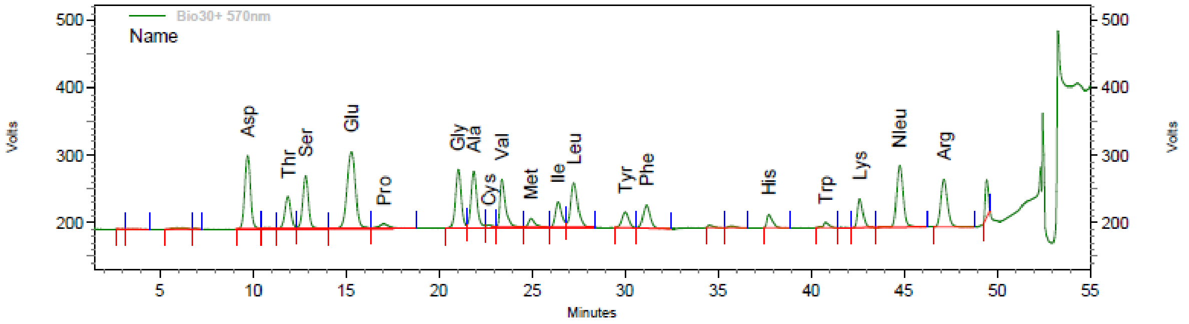

Monoica

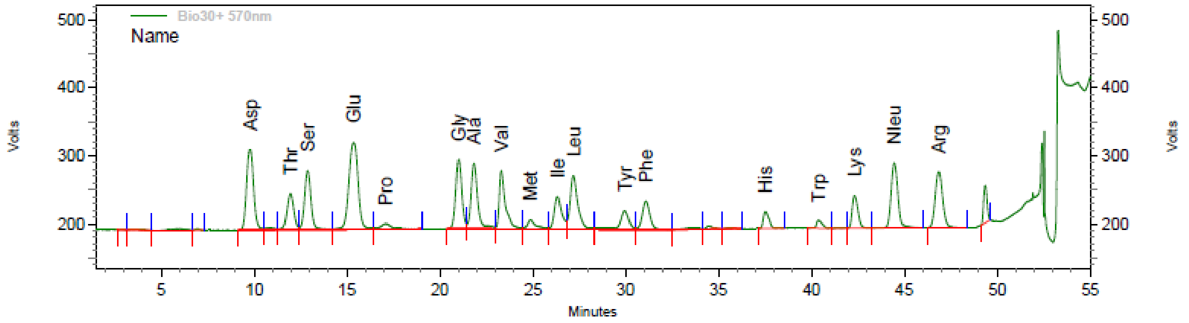

Novosadska

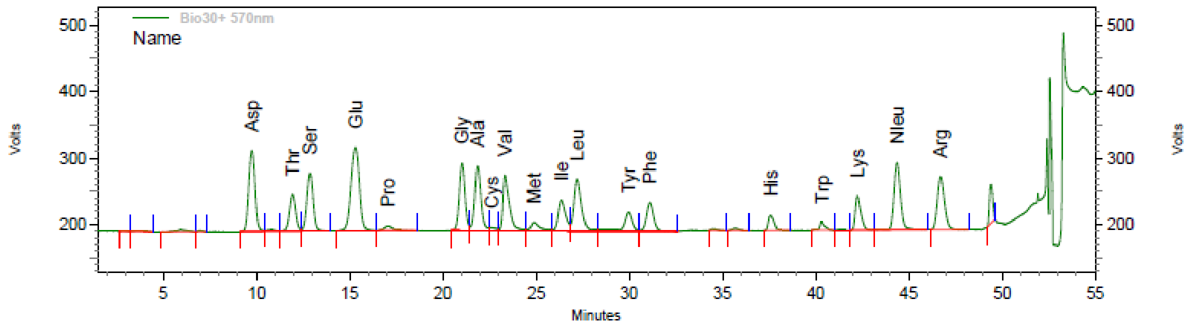

Novosadska+

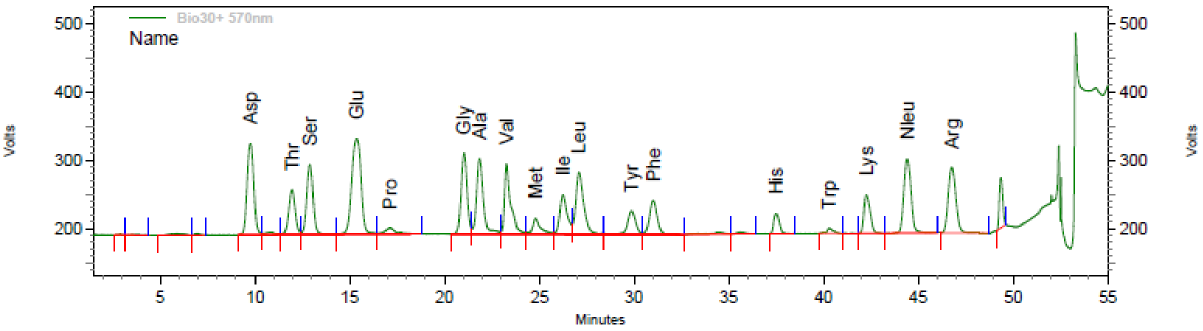

Santhica 23

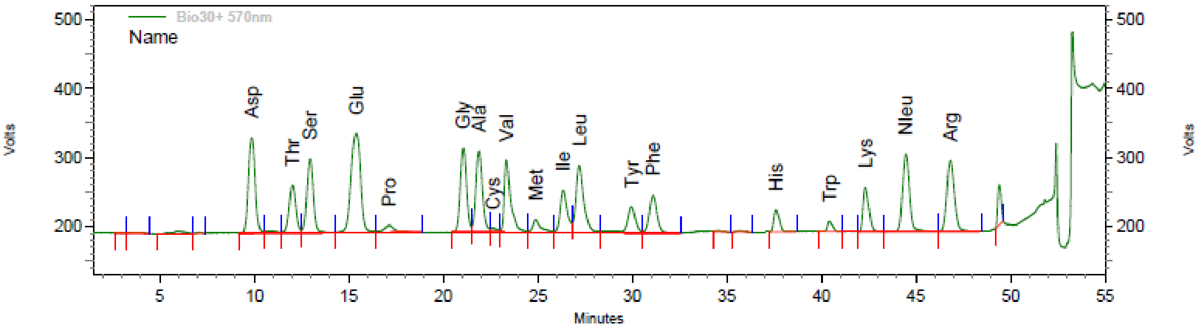

Secuieni jubilee

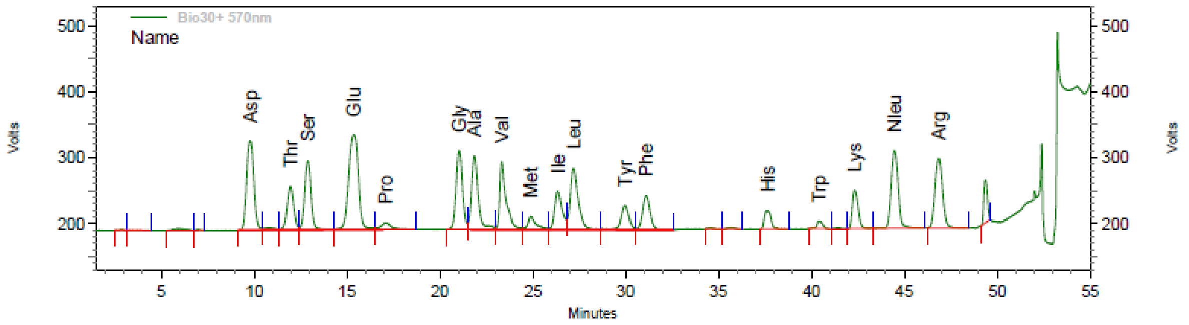

Silesia

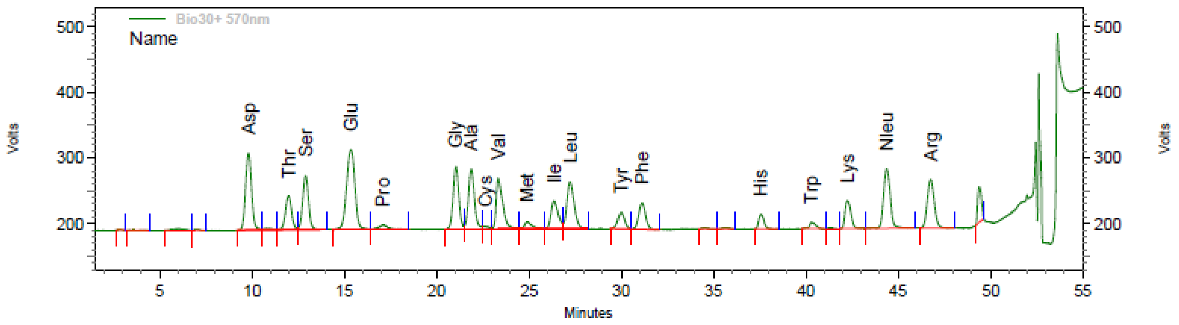

Simba

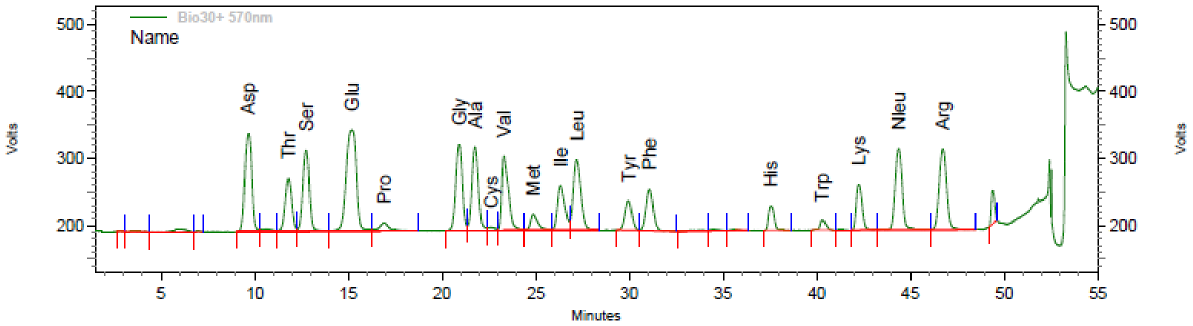

Tiborszallasi

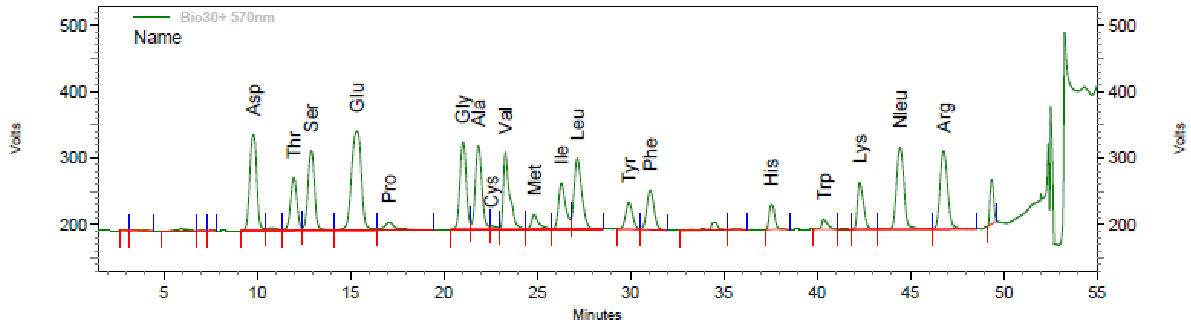

Tisza

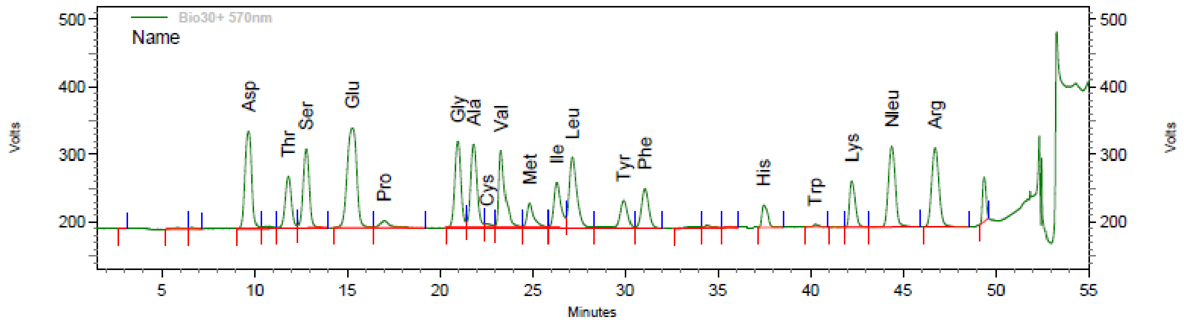

Wojko

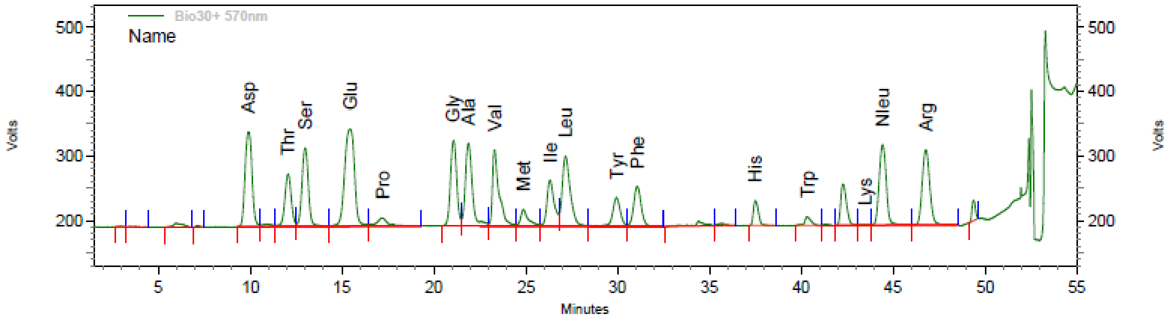

Figure S3. Gas Chromatography-Mass Spectrum Detector chromatograms of cannabinoids from 29 hempseed varieties

Antal

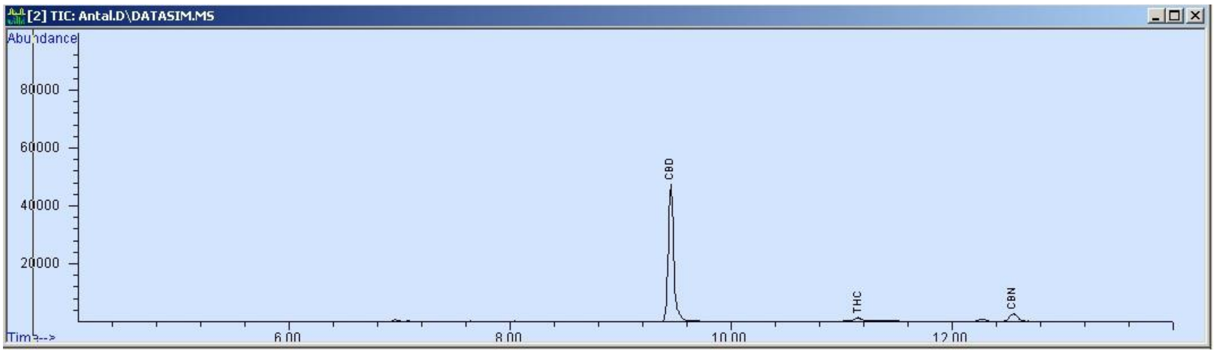

Bacalmas

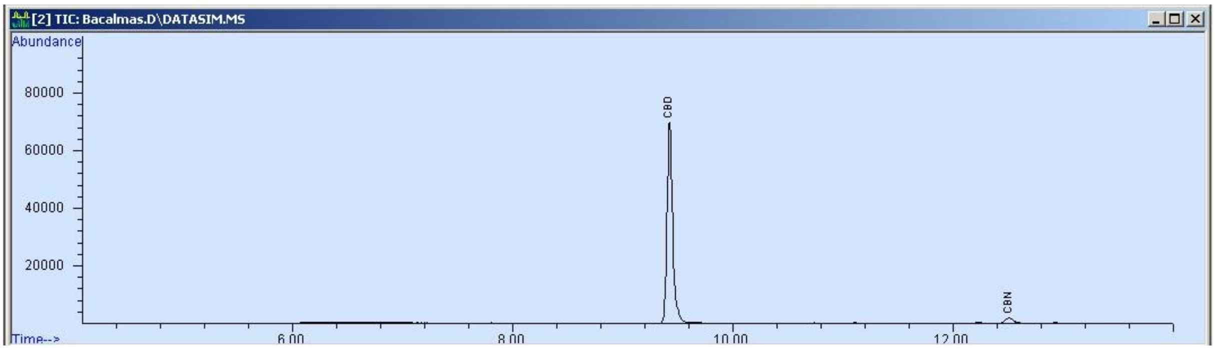

Carmagnola

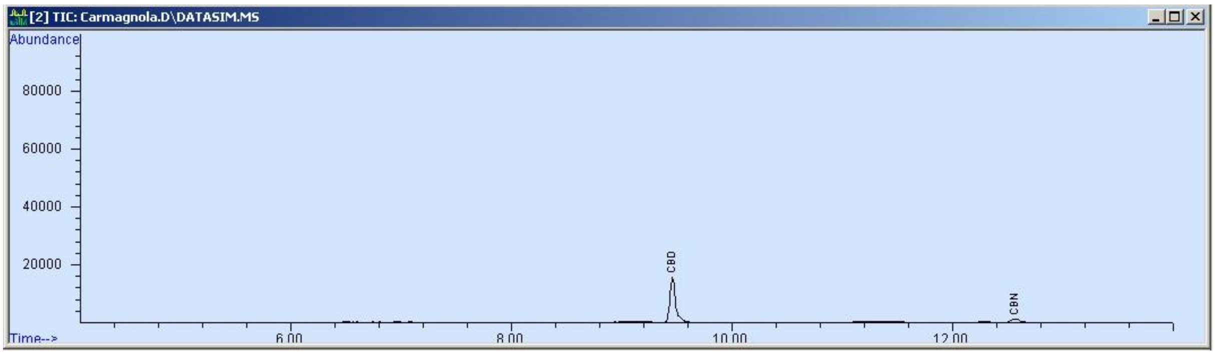

Chameleon

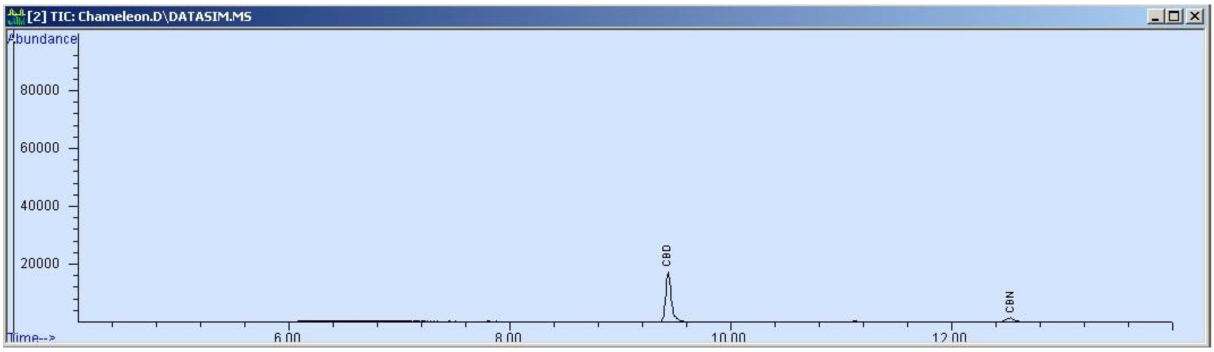

Dioica 88

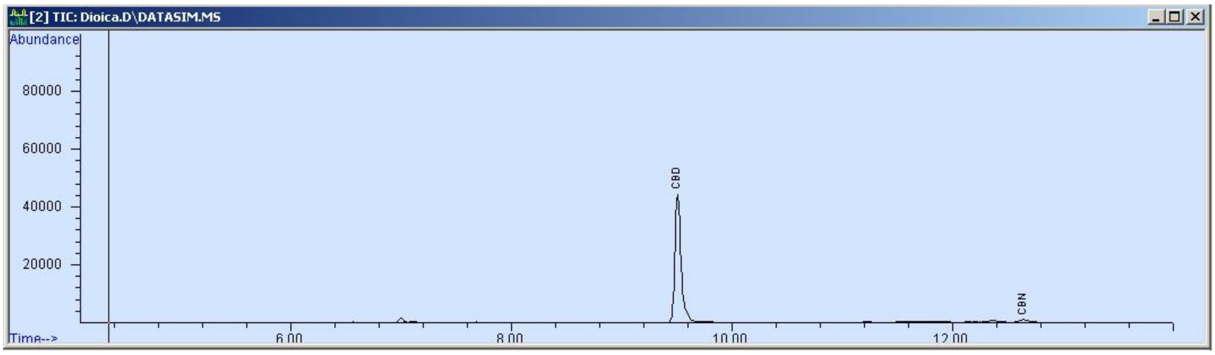

Epsilon 88

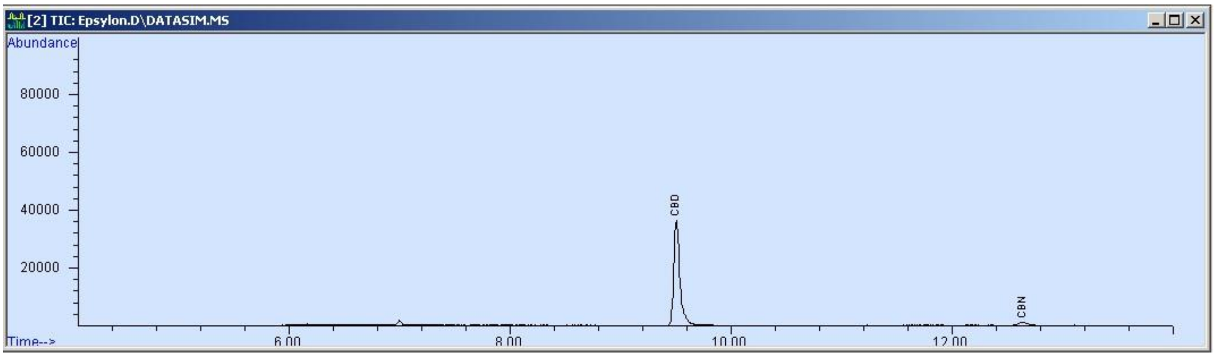

Fedora 17

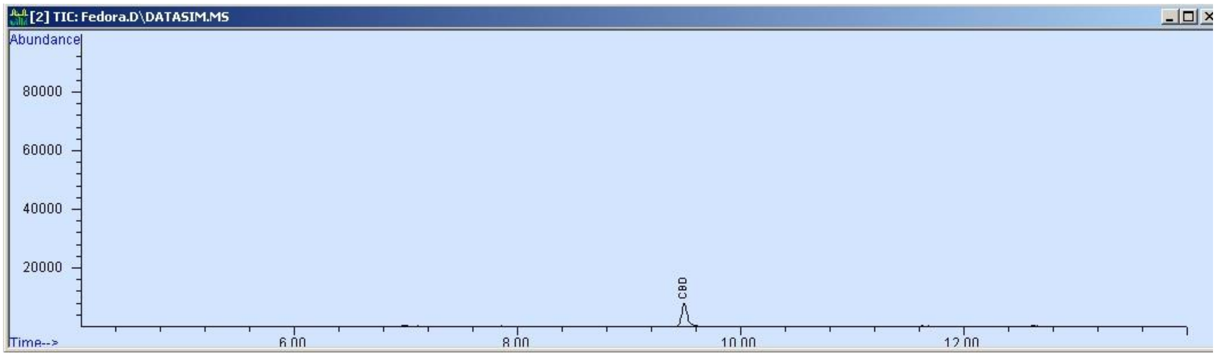

Felina 32

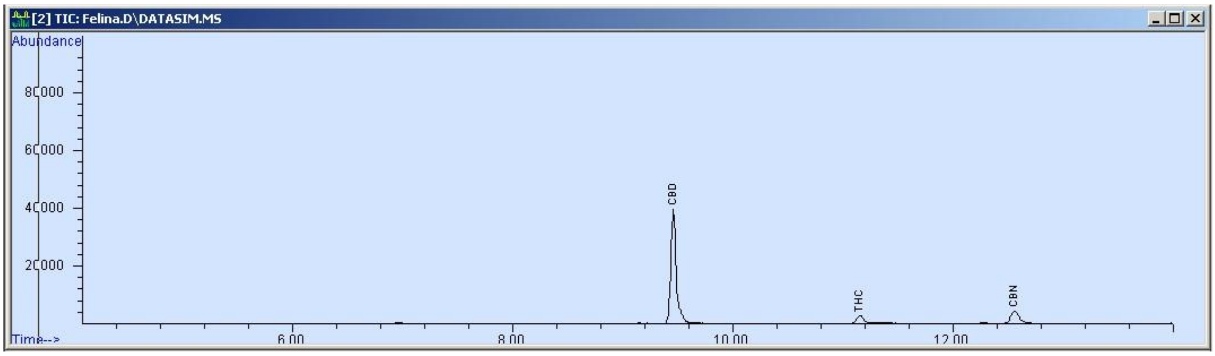

Ferimon FR8194

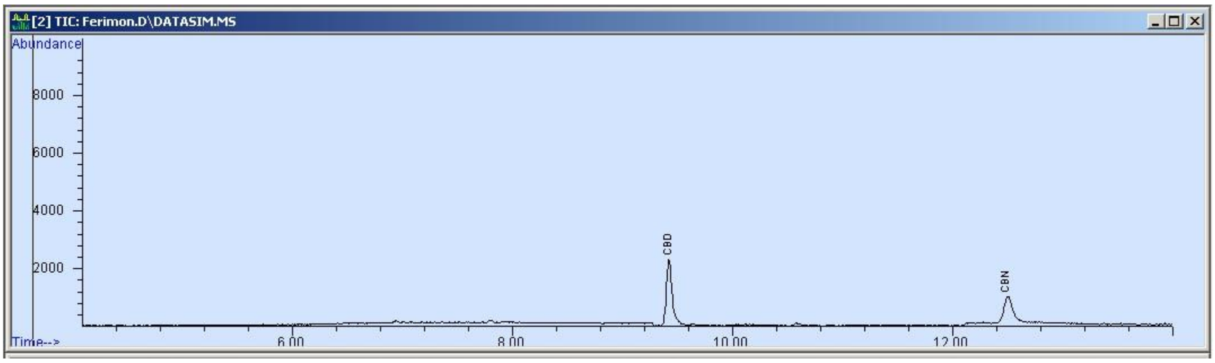

Fibrol

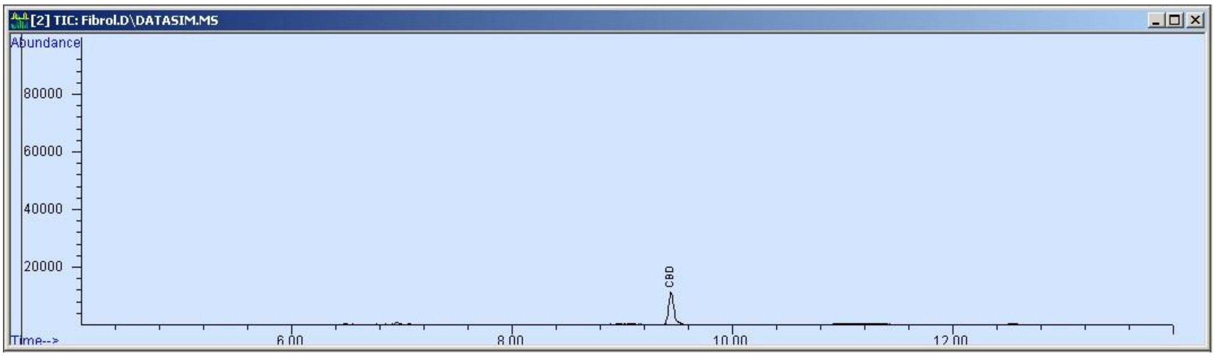

Futura 75

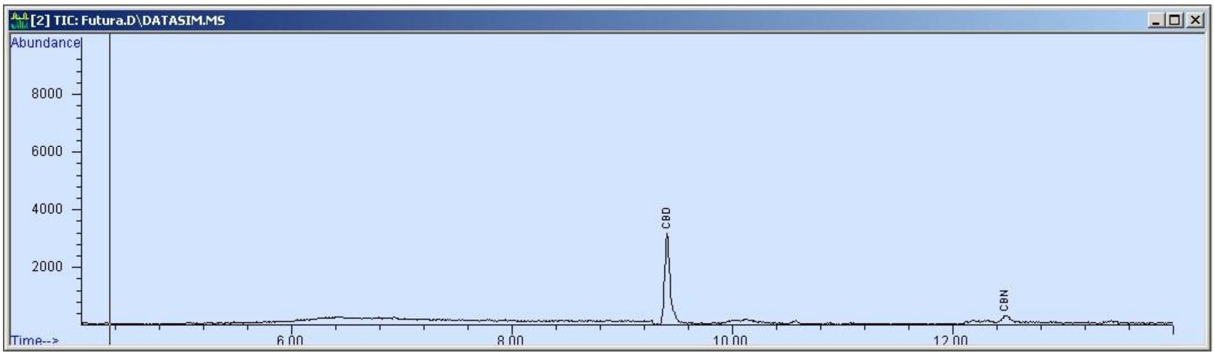

Helena

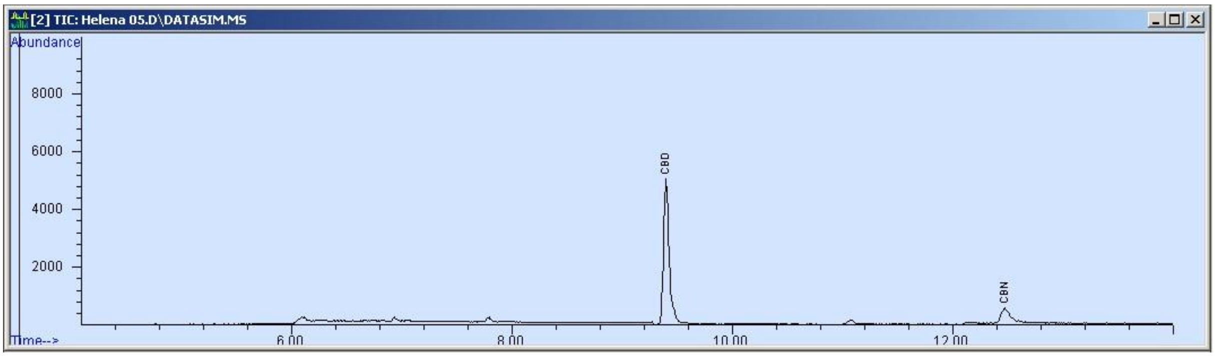

KC Dora

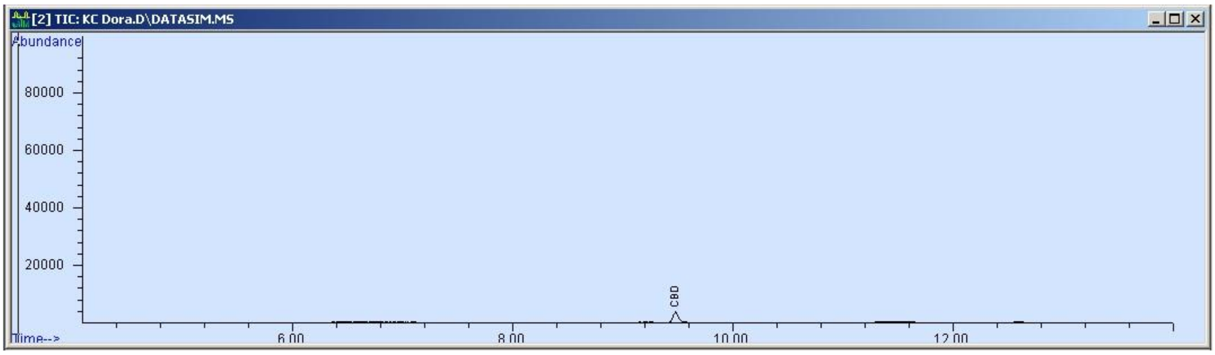

KC Virtus

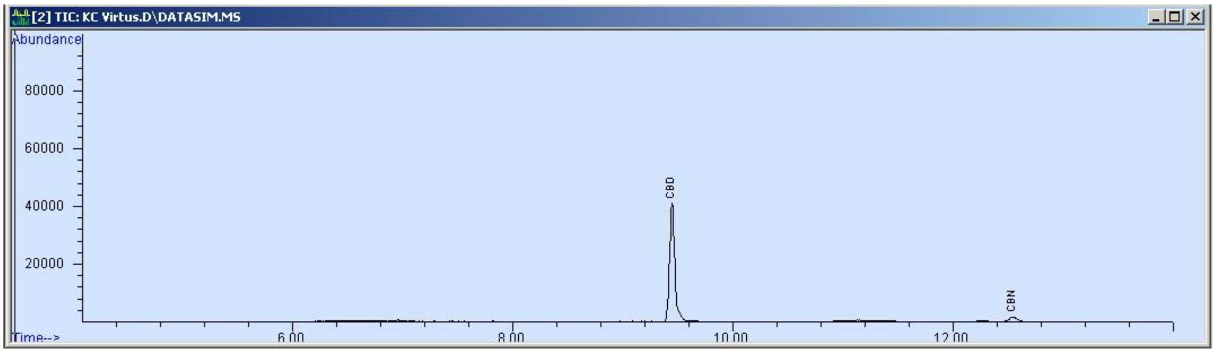

KC Zuzana

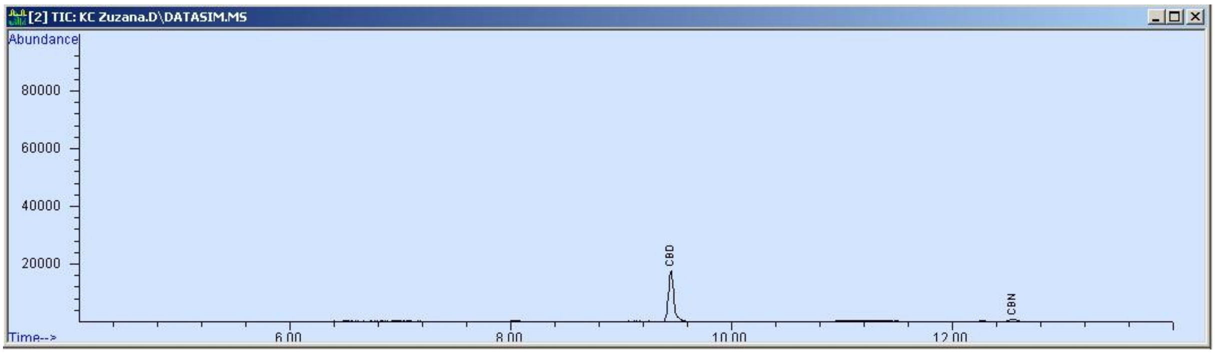

Kina

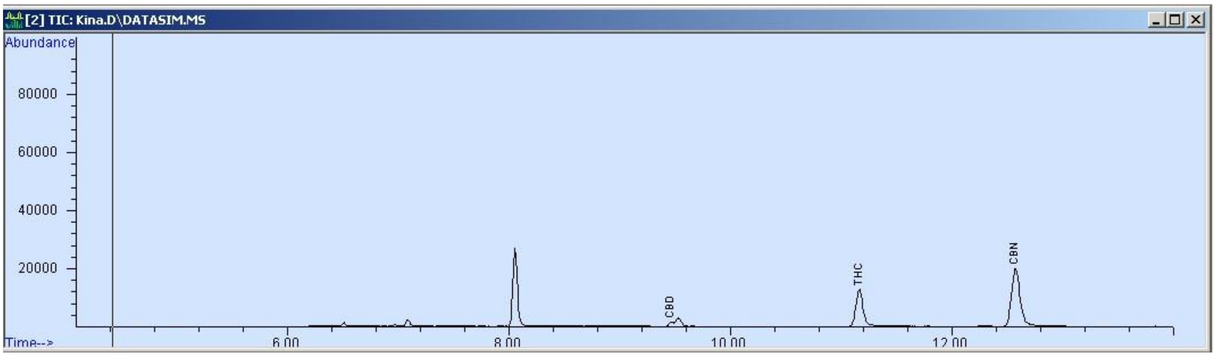

Kompolti

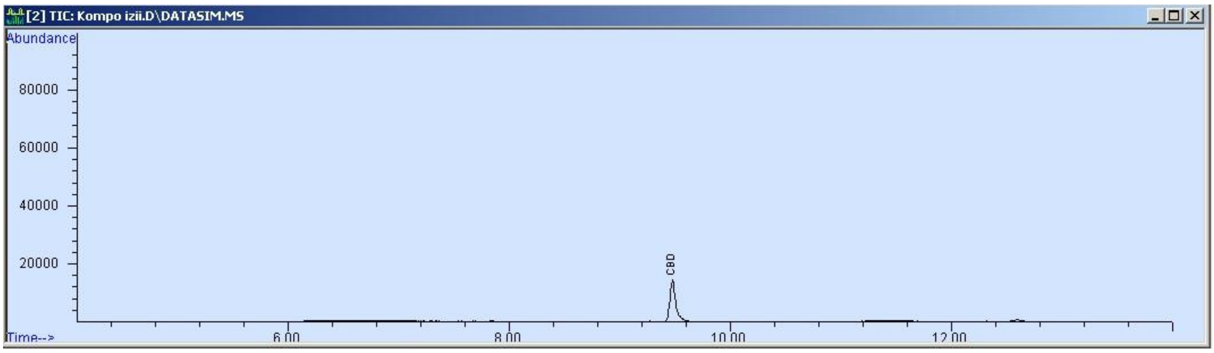

Lovrin110

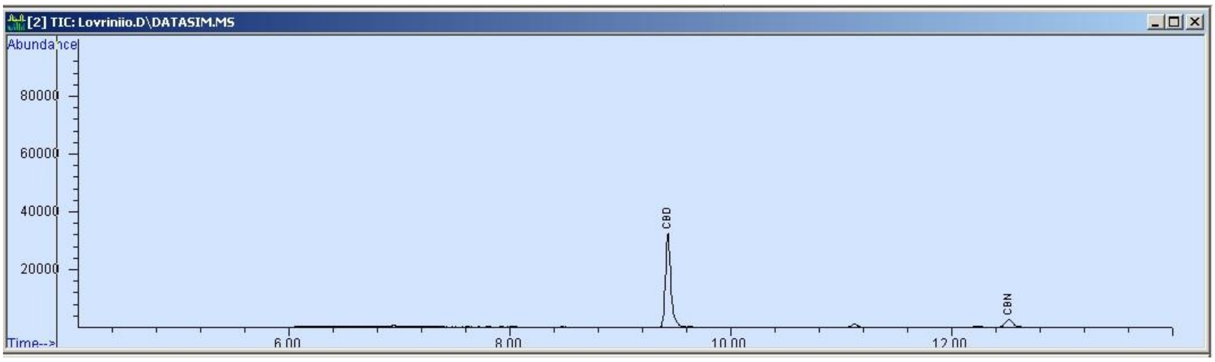

Marina

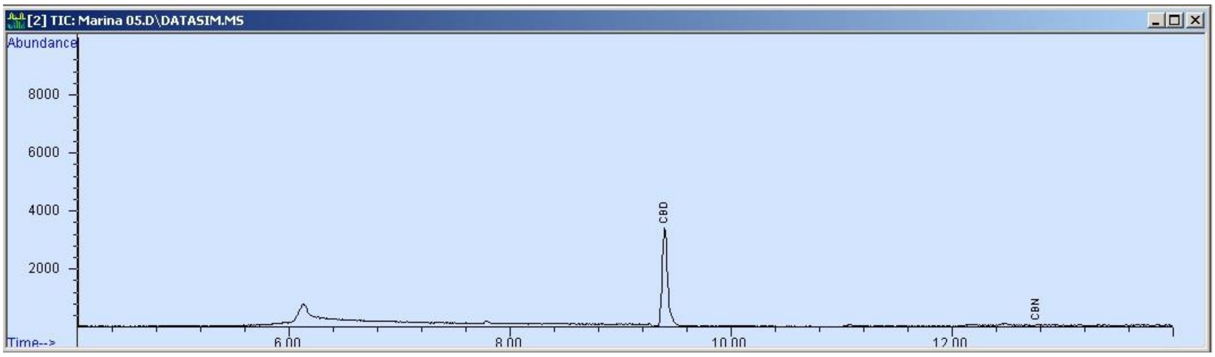

Monoica

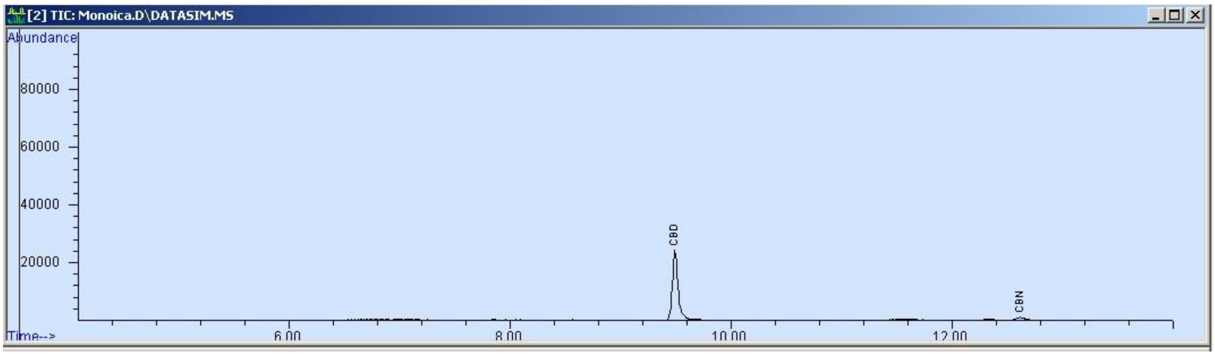

Novosadska

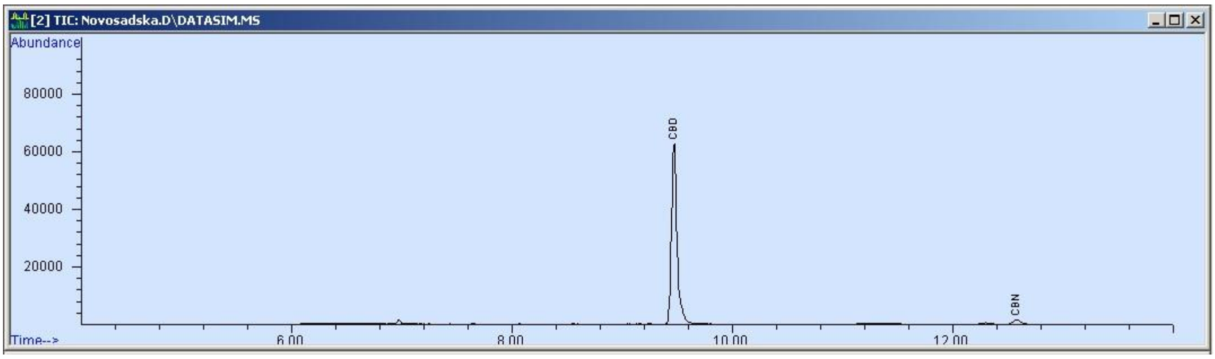

Novosadska +

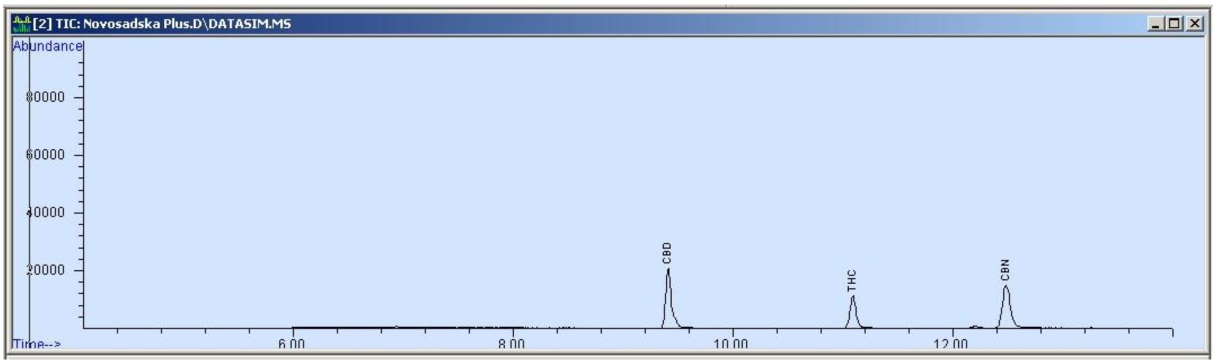

Santhica 23

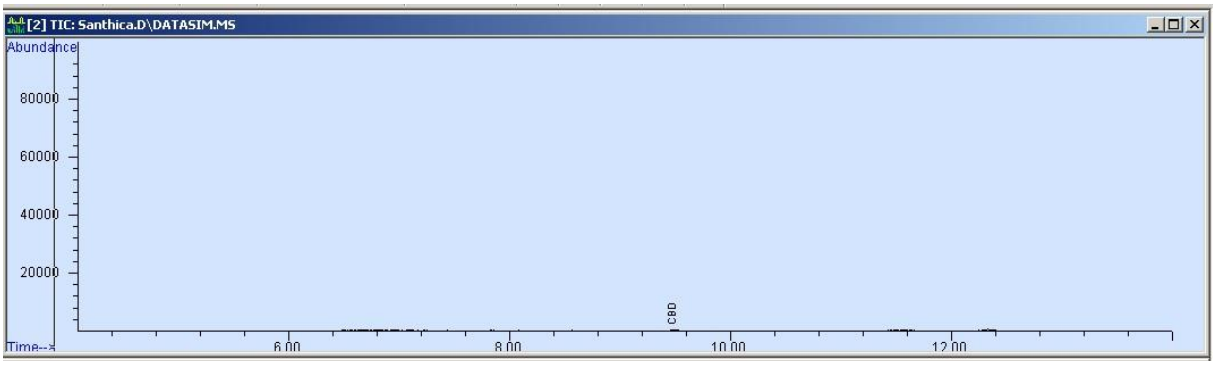

Secuieni jubileu

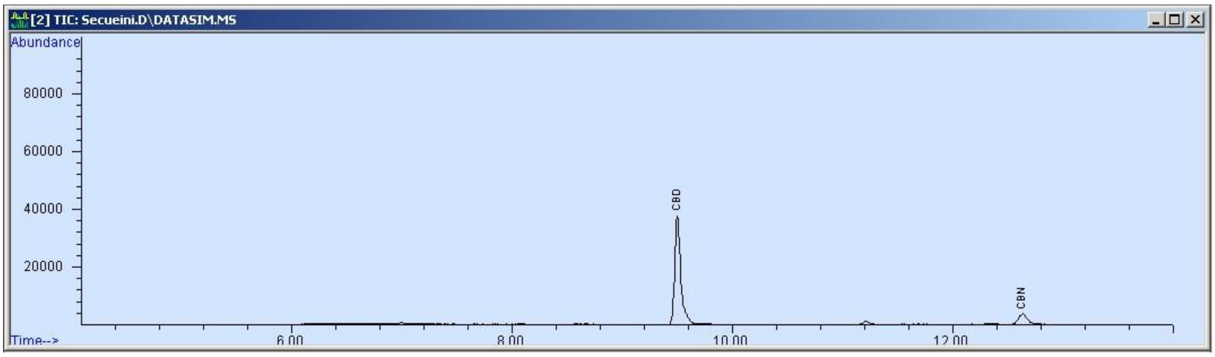

Silesia

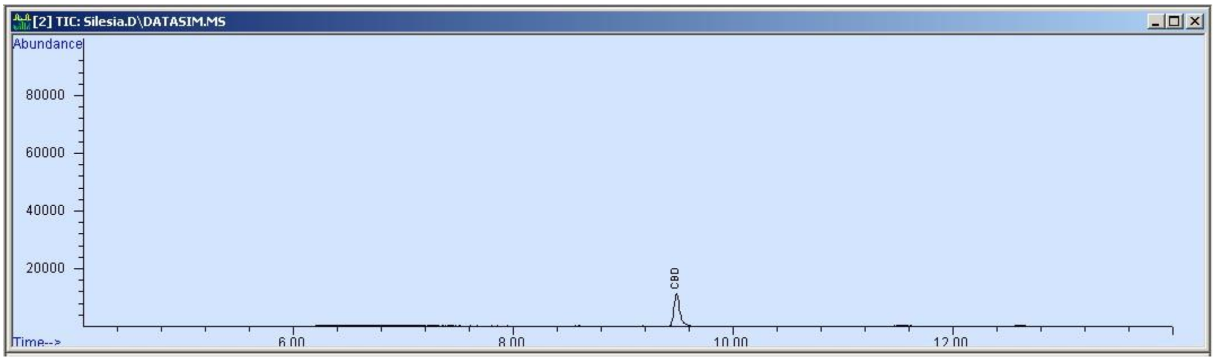

Simba

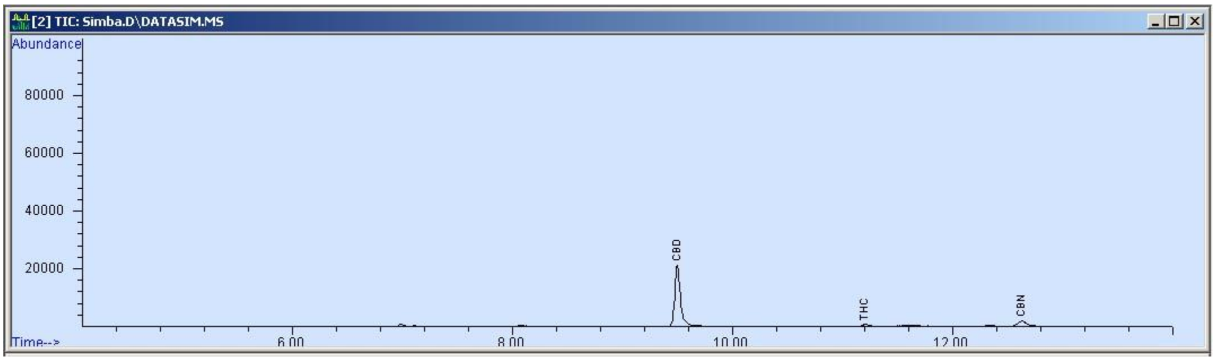

Tiborszallasi

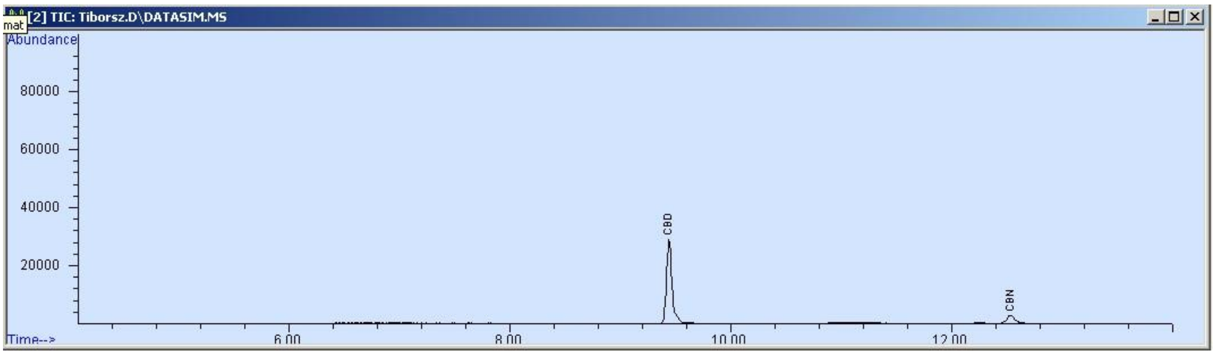

Tisza

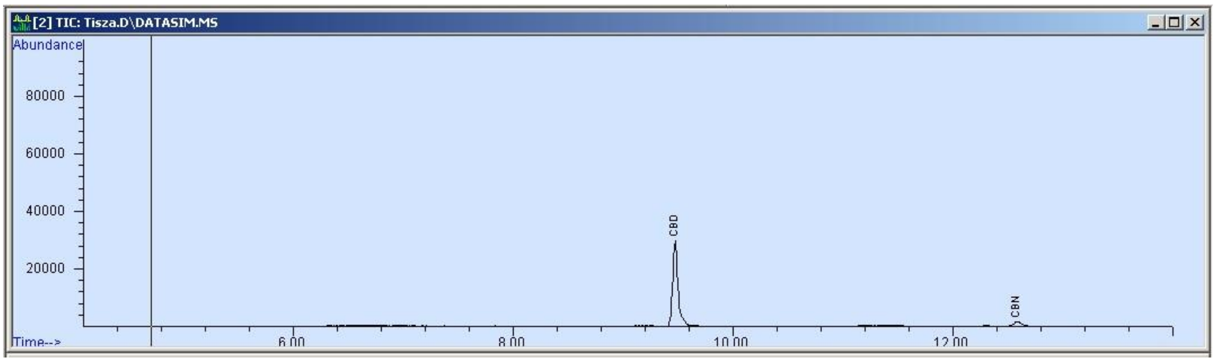
[2] TIC: Tisza.D\DATASIM.MS
Abundance
80000
60000
40000
20000
CBD
CBN
Time-->
6.00
8.00
10.00
12.00

Wojko

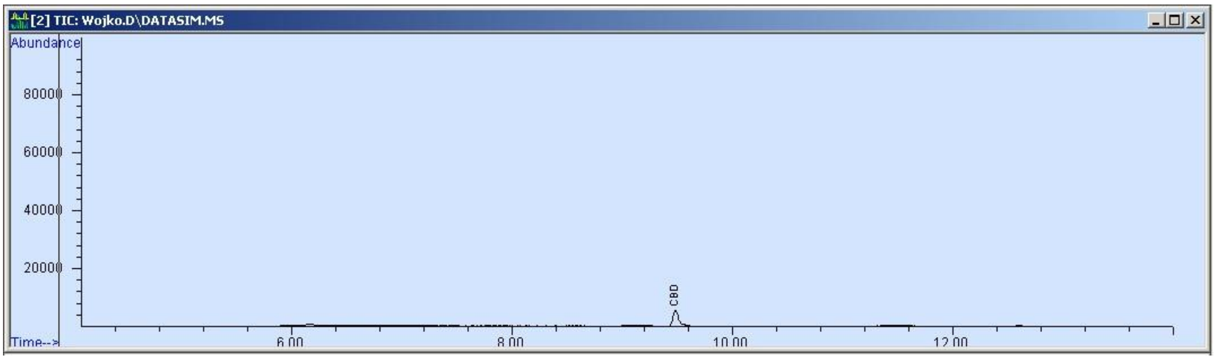
[2] TIC: Wojko.D\DATASIM.MS
Abundance
80000
60000
40000
20000
CBD
Time-->
6.00
8.00
10.00
12.00
